# Characterizing Social Communication Difficulties in Young Children within a Longitudinal Ecological Systems Framework

**DOI:** 10.1007/s10802-025-01308-y

**Published:** 2025-03-24

**Authors:** Samantha C. Perlstein, Wanjikũ F. M. Njoroge, Lauren K. White, Julia Parish-Morris, Alasia I. Williams, Kayla S. Malone, Yuheiry Rodriguez, Sydney Sun, Kate Wisniewski, Ayomide Popoola, Michaela Flum, Sara L. Kornfield, Jakob Seidlitz, Barbara H. Chaiyachati, Ran Barzilay, Raquel E. Gur, Rebecca Waller

**Affiliations:** 1 Department of Psychology, University of Pennsylvania, Stephen A. Levin Building, 425 South University Avenue, Philadelphia, PA 19104 USA; 2 Department of Child and Adolescent Psychiatry and Behavioral Sciences, Children’s Hospital of Philadelphia, Philadelphia, USA; 3 Department of Psychiatry, Perelman School of Medicine, University of Pennsylvania, Philadelphia, USA; 4 PolicyLab, Children’s Hospital of Philadelphia, Philadelphia, USA; 5 Children’s Hospital of Philadelphia and Penn Medicine, Lifespan Brain Institute (Libi), University of Pennsylvania, Philadelphia, USA; 6 Penn Center for Women’s Behavioral Wellness, Department of Psychiatry, Perelman School of Medicine, University of Pennsylvania, Philadelphia, USA; 7 Institute for Translational Medicine and Therapeutics, University of Pennsylvania, Philadelphia, USA; 8 Division of General Pediatrics, Department of Pediatrics, Perelman School of Medicine, Children’S Hospital of Philadelphia, University of Pennsylvania, Philadelphia, USA

**Keywords:** Ecological systems, Maternal bonding, Peripartum risk, Social communication, Toddlerhood

## Abstract

Social communication difficulties are a transdiagnostic risk factor for psychopathology. However, few studies have examined prospective risk for social communication difficulties in young children within an ecological systems framework. Our sample was 251 parent–child dyads assessed during pregnancy, postpartum, and toddlerhood (child ages 1 and 2). We leveraged observer ratings of child behavior, parent-reported questionnaires, and geocoded data. We examined prospective risk factors for social communication difficulties at ages 1 and 2, including at the level of the child (gestational age), family (household income, maternal mental health, maternal-child bonding), and neighborhood (neighborhood resources). Fewer neighborhood resources were associated with more social communication difficulties at age 1, but only among dyads with impaired maternal bonding. Lower gestational age, lower household income, and impaired maternal bonding were associated with more child social communication difficulties at age 2. Fewer neighborhood resources were also related to more social communication difficulties at age 2, specifically among families with low household incomes. Findings provide insight into families who may benefit from early intervention to reduce transdiagnostic risk for child psychopathology across ecological systems, including efforts to target maternal bonding and poverty.

**Supplementary Information:**

The online version contains supplementary material available at 10.1007/s10802-025-01308-y.

The prevalence of psychiatric disorders in early childhood is 20% (Vasileva et al., 2021). However, psychiatric symptoms change across development and symptoms can emerge after the etiological processes central to psychopathology have begun to unfold (Wakschlag et al., 2019). Thus, we need to identify children at risk for developing psychiatric disorders as early as possible and target preventative intervention strategies more effectively. In the current study, we examined early risk factors for social communication difficulties, an important transdiagnostic risk factor for psychopathology (Mikami et al., 2019; Perlstein et al., 2022). Guided by an ecological systems framework, we tested multiple and interacting influences, including child risk (e.g., gestational age), proximal factors in the “microsystem” (e.g., parenting), and distal factors in the “exosystem” (e.g., neighborhood), as well as their interplay via the “mesosystem” (e.g., parenting in the context of fewer neighborhood resources) (Fig. 1; Bronfenbrenner, 1992).

**Fig. 1:**
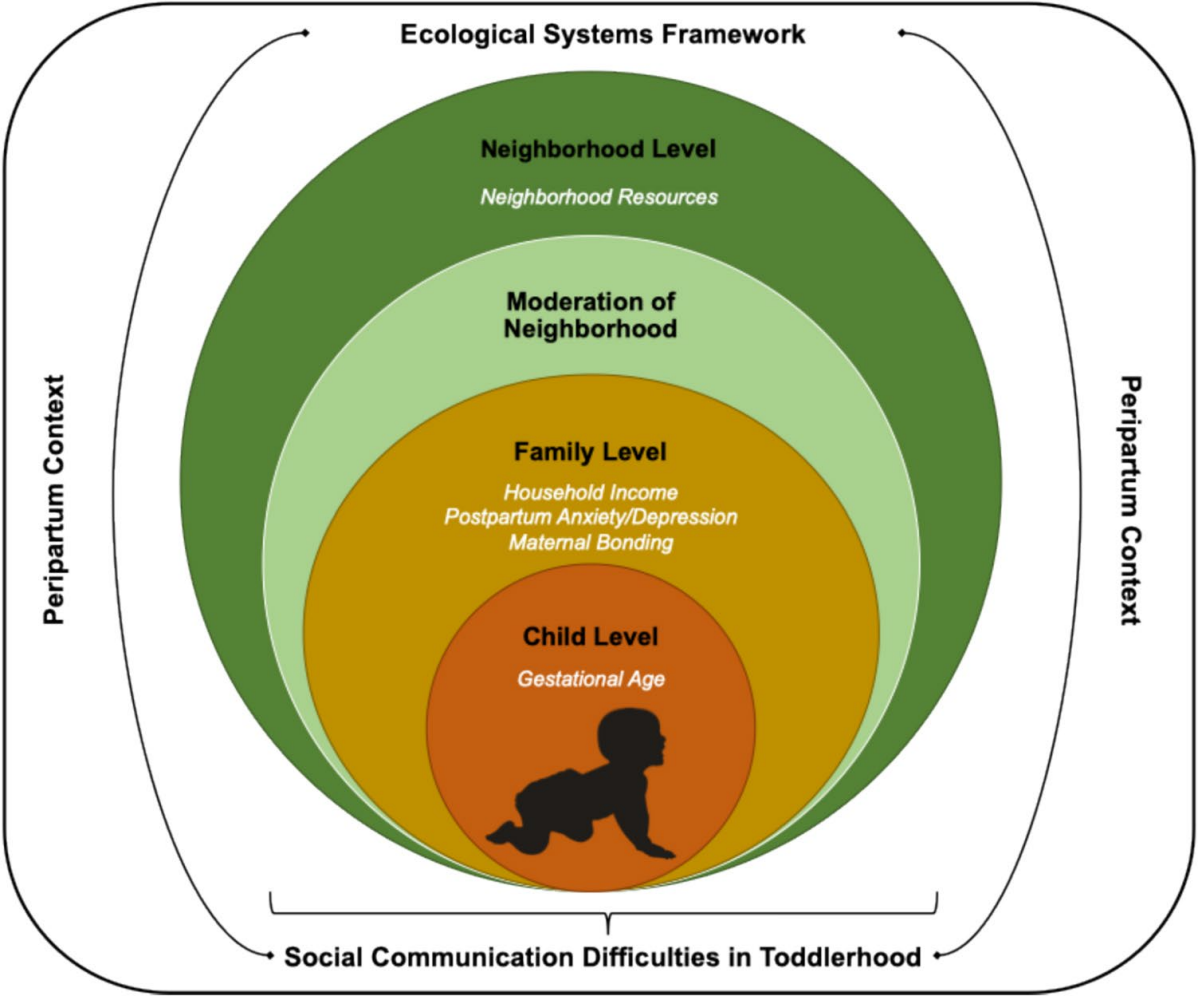
Ecological Systems Framework to Examine Peripartum Risk for Social Communication Difficulties in Toddlerhood

Social communication is the ability to initiate and respond appropriately to others in ways that promote social connections, thus adaptively centering an individual within their social environment (Prizant & Wetherby, 1990). In late infancy and toddlerhood, social communication is evidenced by a receptiveness to and the production of social information, including both verbal and non-verbal signals (Uljarević et al., 2021). Not surprisingly, difficulties with social communication are associated with psychopathology in children, including depression in 3–6-year-olds (Meagher et al., 2009), disruptive behavior disorder in 6–13-year-olds (Donno et al., 2010), and callous-unemotional traits in 2–3-year-olds (Perlstein et al., 2022). Social communication difficulties in early childhood are also known to index risk for the development of autism spectrum disorder (Costescu et al., 2022). Toddlerhood is a critical period for socioemotional development when language production rapidly increases, alongside early affiliative behaviors (e.g., positive vocalizations, social touch) that promote interpersonal connection (Shulman, 2016). However, less is known about the interplay of different ecological systems on social communication development prior to toddlerhood.

In terms of risk factors, prior research has consistently linked child and family (i.e., microsystem) factors assessed in the perinatal period to child socioemotional and behavioral difficulties. For example, preterm birth was linked to disruptions in cortical development and brain connectivity (Ment & Vohr, 2008) and meta-analytic work points to lasting effects of preterm birth on social behavior (Aarnoudse-Moens et al., 2009) and language development (van Noort-van der Spek et al., 2012). Prenatal maternal anxiety is also related to risk for poor child social-emotional outcomes in toddlerhood (Blair et al., 2011). Moreover, maternal postpartum depression has been linked to child social difficulties, thought to arise from disruptions in mother–child interactions and reductions in the types of responsive parenting behaviors that would otherwise promote child self-regulation (Beck, 1998). Notably, even among mothers without prenatal or postpartum mental health concerns, early disruption to mother–child interactions is linked to worse child outcomes (Barwick et al., 2004). Conversely, in a longitudinal study of 97 healthy mothers, positive maternal-child bonding was related to increased social competency at age 5 (Joas & Möhler, 2021). However, few studies have tested how impaired early maternal bonding relates to child social communication difficulties over and above maternal postpartum depression. Prior studies have also relied on small convenience samples that lack variability in psychiatric symptoms or that include few participants from more socioeconomically disadvantaged communities.

Within the exosystem, children living in low resourced neighborhoods, characterized by significant family poverty, unemployment, crime, and environmental toxicant exposure are at risk for poor physical and mental health outcomes, as well as low academic attainment and unemployment (Bhutta et al., 2023). Such neighborhood influences operate in concert with more proximal family-level factors, including parenting (Hyde et al., 2020) and the parent–child relationship (Ho et al., 2022) (i.e., mesosystem). In contrast, children living in higher resourced neighborhoods, characterized by more families in employment who own their own homes (Leventhal & Brooks-Gunn, 2003), have better academic attainment and lower risk for psychopathology, over and above the individual income of families (Leventhal et al., 2006). To best capture the impact of neighborhood resources and influences, the Childhood Opportunity Index (COI) characterizes both traditional neighborhood metrics (e.g., median income, unemployment) and other neighborhood attributes (e.g., childcare facilities, green space, air quality) that are salient to family well-being and child development (Acevedo-Garcia et al., 2014; Aris et al., 2021). Lower COI scores were linked to externalizing problems in 9–10-year-old children (Beyer et al., 2024), worse cardiometabolic health in 7–13-year-old children (Aris et al., 2021), and increased use of urgent care facilities for children with medical complexities (Fritz et al., 2022). Importantly, while environmental adversity assessed during pregnancy (e.g., poverty, discrimination) has been linked to infant brain-behavior development (Monk et al., 2019), no studies have tested whether COI or other measures of neighborhood resources assessed during pregnancy relate to social communication difficulties in the first years of life. Moreover, no studies have adopted an ecological systems framework to examine the interaction of neighborhood resources with other child and family level risk factors (e.g., gestational age, maternal depression) in relation to risk for social communication difficulties (i.e., mesosystem) (Bronfenbrenner, 1992).

Finally, studies of structural racism demonstrate that many neighborhood level stressors disproportionally impact racially minoritized families in the United States (Paradies et al., 2014). Prior research also indicates that structural racism (i.e., practices that maintain or exacerbate inequalities in opportunities across racial/ethnic groups; Paradies et al., 2014) has lasting effects on brain development through stress-related epigenetic process and complex trauma (Njoroge et al., 2023). However, few studies have examined whether child, family, and neighborhood level factors differentially relate to child social communication difficulties for families experiencing greater discrimination.

Thus, in the current study, we examined risk factors for social communication difficulties using a multi-method approach and guided by an ecological systems framework. Under our first aim, we derived a multi-method factor to assess social communication difficulties at age 2, combining reports from a primary and secondary caregiver, observer ratings of child social engagement during parent–child interaction tasks, and an objective metric of child verbal expressivity transcribed from social interactions. For our second aim, and to better inform prevention efforts, we investigated risk pathways to social communication difficulties that emerge in the perinatal period. Consistent with an ecological systems framework, we examined interacting risk factors at the level of the child (gestational age), family (household income, maternal depression and/or anxiety, maternal bonding), and neighborhood (COI), assessed across the peripartum period. In a subsample of dyads, we examined similar risk pathways in relation to observed social communication difficulties at age 1. Finally, we examined risk pathways to social communication difficulties at age 2 in the context of systemic racism, examining maternal perceptions of discrimination in the postpartum period. Our overarching goal was to elucidate child, family, and neighborhood factors that contribute to early social communication difficulties with the goal of improving future assessment methods and providing targets for preventive interventions to be implemented across the peripartum, infancy, and toddlerhood periods.

## Methods

### Participants and Procedures

Participants were *N* = 251 mother–child dyads (child age, *M* = 26.43 months, *SD* = 0.95) from the Prenatal to Preschool (P2P) study (Njoroge et al., 2023), a sub-study of a larger perinatal cohort (Waller et al., 2022). Prospective data were available from pregnancy (time 1), childbirth (time 2), 10–15 weeks postpartum (time 3), child age 1 (time 4), and child age 2 (time 5; see Supplement and Figure S1). At time 5 (child age 2), mothers identified their race as White (*n* = 129 51.4%) or Black or African American (*n* = 122, 48.6%). Six individuals also self-identified as Latino/a/e/x (2.4%). Data were collected using questionnaires and from an online visit (time 5). Questionnaire data were also available from secondary caregivers for 86% of the sample (*n* = 217), of whom 86% (*n* = 186) identified as fathers. A subset (*n* = 163) of the 251 dyads had observational data from a separate online visit at age 1 (child age, *M* = 12.95 months, *SD* = 0.95; White, *n* = 82 50.3%; Black or African American, *n* = 81, 49.7%). Of the 251 dyads with data from age 2, 61% had data available from all five time points (*n* = 153; pregnancy, delivery, postpartum, year 1, and year 2 visits), 32% had 4 time points (*n* = 81), and 7% had 3 time points (*n* = 17). Data were available from all participants at time 1, 2, and 5. Using full information maximum likelihood estimation with robust standard errors (Enders & Bandalos, 2001), models addressing our study aims leveraged the full P2P study sample of *N* = 251 at time 5 (see Analytic Strategy and Supplemental Methods and Table S1 for additional information on data availability). Participants provided online informed consent with an electronic signature. The study was approved by the Institutional Review Boards of the Children’s Hospital of Philadelphia and the University of Pennsylvania.

### Measures

We combined parent report data collected during pregnancy (time 1), medical record data from childbirth (time 2), parent reported data from 10–15 weeks postpartum (time 3), observer ratings of parent–child interactions for a subsample of families who completed an online visit at age 1 (time 4), and parent report, secondary caregiver report, and observer ratings of parent–child interactions from an online study visit at age 2 (time 5) (Table S2).

#### Social Communication Difficulties (age 2)

We derived a multi-method social communication difficulties factor that combined parent and secondary caregiver report measures, observer ratings of child social engagement from three parent–child interaction tasks, and child verbal expressivity from transcribed child verbal output during the visit. First, we used parent and secondary caregiver reports on items that overlapped conceptually with the construct of social communication difficulties, including 10 items from the Ages and Stages Questionnaire (ASQ; Squires et al., 2015) and 7 items from the preschool version of the Child Behavior Checklist (CBCL; Achenbach & Rescorla, 2000). Items were rated on a 3-point Likert scale (0 = rarely/never, 1 = sometimes, 2 = often/always; see Table S3 for list of items). Items from the ASQ were reversed scored. Internal consistency was acceptable for both mother (ASQ, α = 0.80; CBCL, α = 0.76) and secondary caregiver (ASQ, α = 0.82; CBCL, α = 0.84) report. To aid model estimation, we combined reports on the ASQ and CBCL for mothers (17-items, α = 0.87) and secondary caregivers (17-items, α = 0.89).

Second, we assessed social communication difficulties using observer ratings of child social engagement during online study visits at age 2, which were recorded. Social engagement captured the child initiating and/or maintaining social interactions with the parent or communicating positive regard/affect to the parent (e.g., smiles, vocalizations). We rated child social engagement during 3 tasks adapted from the Three Bags procedure (see Supplemental Methods), each lasting 2 min: free play (dinosaur toy), a structured task (cube puzzle, with parent instructed to provide the child with assistance as needed), and a storybook reading task (parents read a wordless book with their child) (Njoroge et al., 2023; Vandell, 1979; Figure S2). A team of three trained researchers rated interactions, with coders required to code 10 or more videos to establish reliability before coding independently. Weekly meetings were held by the coding team to maintain fidelity. Coders watched tasks 3 times before rating child social engagement from 1 (very low) to 7 (very high). Inter-rater reliability was calculated on a random 15% of videotapes and was high across tasks (*range, ICC* = 0.76–0.91). Scores across tasks correlated (*range, r* = 0.51–0.73, *p* < 0.001) and were combined into a single measure to reflect child social engagement across contexts. For a subset of families with available data at age 1 (*n* = 163), we derived a similar observed social communication difficulties measure based on observer ratings of child social engagement during two parent–child interaction tasks completed during an online study visit that was recorded. At age 1, we focused on a free play task (parent and child played with a novel rattle) and a storybook reading task (parents read a wordless book with their child) (Vandell, 1979). Training, reliability, and scoring procedures were similar to those described with inter-rater reliability, calculated on a random 20% of videotapes, high for both the free play (ICC = 0.84) and reading (ICC = 0.80) tasks. Scores were moderately correlated (*r* = 0.39, *p* < 0.001) and, as at age 2, were combined into a single measure to reflect age 1 social engagement across contexts. At both ages 1 and 2, we re-coded scores such that higher ratings indexed lower social engagement (i.e., social communication difficulties).

Third, we derived an objective metric of social communication difficulties at age 2 using child expressed language during the online visit. As described above, parent–child dyads completed three tasks, as well as a clean-up task (parents and children played with a farmer’s market basket toy and then had to unexpectedly return all items to the basket; Figure S2). To derive a metric of child language expression, a team of three researchers transcribed child verbal expressions from recorded interactions for all tasks using a transcription manual to ensure consistency. Transcription data were uploaded into the Linguistic Inquiry and Word Count (LIWC) program (Boyd et al., 2022). For our analysis, we used total child word count divided by the length of the interaction tasks (length [minutes], *M* = 14.88, *SD* = 2.93, *range* = 8–41). We double-transcribed 20% of videos, with high inter-rater reliability between transcriptions (ICC = 0.85). See Supplemental Methods for more information.

### Child-Level Risk Factors

#### Gestational Age (time 2)

We used medical record data to capture gestational age in weeks (M = 38.78, SD = 1.53, range = 29–41).

### Family Level Risk Factors

#### Household Income (time 3)

Annual household income was assessed using parent report on a single questionnaire item: 1 = less than $20,000 (*n* = 25, 10%); 2 = $20,000 to $60,000 (*n* = 45, 18%); 3 = $60,000 to $100,000 (*n* = 37, 15%), and 4 = more than $100,000 (*n* = 115, 46%).

#### Maternal Mental Health (time 3)

We assessed postpartum depression using 9 items of the 10-item Edinburgh Postnatal Depression Scale (EPDS; Cox & Holden, 2003), with item 10 (i.e., assessing self-harm) excluded given difficulties associated with monitoring self-harm online. Items were rated on a 4-point scale (0 = not at all to 3 = all the time; α = 0.88). Due to shared method variance (*r* = 0.34, *p* < 0.001) between continuous mother-reported scores on the EPDS and the maternal bonding questionnaire described below, we used established cut-off scores of > 10 to create a binary indicator that distinguished mothers who met clinical screening thresholds for postpartum depression (*n* = 48, 19.1%; Levis et al., 2020). See Supplemental Results for more information. We assessed clinically significant symptoms of general anxiety using the 7-item GAD-7 (Spitzer et al., 2006), with items rated on a 4-point Likert scale (1 = not at all to 4 = nearly every day) and a cut off score of > 10 (*n* = 22, 8.8%; Plummer et al., 2016) (α = 0.92). We created a binary indicator (0 = absent, 1 = present) to indicate clinically significant levels of postpartum depression and/or anxiety (*n* = 48, 19.1%).

#### Impaired Maternal bonding (time 3)

Impaired maternal bonding was assessed using the 12-item impaired bonding subscale (e.g., ‘‘I feel close to my baby”, “I love my baby to bits”, “My baby irritates me”, “My baby cries too much”) from the Postpartum Bonding Questionnaire (PBQ; Brockington et al., 2006), with items rated on 6-point scale (0 = always, 5 = never). As relevant, items were reverse scored, such that higher total scores derived by summing the 12 items indexed higher levels of impaired maternal bonding (α = 0.80).

#### Perceived Discrimination (time 3)

Maternal Perceived Discrimination was assessed using the 10-item Everyday Discrimination Scale (EDS; Williams et al., 1997), with items (e.g., “you receive poorer service than other people at restaurants or stores”) rated on a 6-point scale (1 = Never, 6 = Almost every day; α = 0.94).

### Neighborhood Level Risk Factors

#### Neighborhood Resources (time 1)

We derived a latent factor of neighborhood resources combining data from four geocoding-based assessments from pregnancy ZIP codes. First, we used a neighborhood socioeconomic status (SES) factor derived from census-based geocoding of neighborhood-level variables (e.g., % in poverty, % married, median family income) (Njoroge et al., 2023). Second, we included the COI, which has 29 indicators that map onto three indices: 1) neighborhood social structure/economic resources (e.g., neighborhood poverty); 2) quality of environment and resources for healthy living (e.g., air pollution, green space); and 3) educational resources (e.g., early child education centers) (Noelke et al., 2021; Supplemental Methods). We conducted confirmatory factor analysis in Mplus (Muthén & Muthén, 1998) specifying the four indicators to load onto an overarching latent factor of neighborhood resources (Table S4).

#### Covariates

Covariates were child age at the time of assessment, child sex, parity when target child was born (i.e., prior versus no prior children), minoritized race of mothers (i.e., Black and/or Latino/a/e/x), and maternal age when target child was born. For supplemental analyses we examined all findings controlling for mother-reports of pandemic related worries in pregnancy and observed sensitive parenting behaviors at ages 1 and 2 (see Supplemental Methods).

### Analytic Plan

All analyses were conducted in Mplus version 8 (Muthén & Muthén, 1998). To examine our first aim, we tested the fit of measurement model to derive a multi-method social communication factor at age 2. We combined mother- and secondary caregiver-report on the ASQ and CBCL, observed ratings of child social engagement, and child verbal expressivity. Model fit was evaluated using standard criteria for chi-square, Comparative Fit Index (CFI), Tucker-Lewis Index (TLI), and the Root Mean Square Error of Approximation (RMSEA) (Hu & Bentler, 1999). To test our second aim, we estimated path models regressing the latent social communication factor at age 2 onto child gestational age, household income, postpartum maternal depression/anxiety, maternal bonding, neighborhood resources, and demographic covariates. Next, we added product terms to our regression model representing the mesosystem, including between neighborhood resources and family income, maternal depression/anxiety, and maternal bonding. We also examined the cross-sectional relationship between observed sensitive parenting and child social communication at age two and re-ran all models after controlling for sensitive parenting. For a subset of families with available data at age 1 (*n* = 163), we re-ran models specifying the observed social engagement difficulties at age 1 as the dependent variable in models with main and interaction terms as described above. We probed significant interactions at mean levels and 1 *SD* above and below the mean (Aiken et al., 1991). Finally, in supplementary analyses, we examined risk pathways to social communication difficulties at age 2 in the context of systemic racism. Specifically, we included maternal reports of perceived discrimination in the postpartum period in models and examined interactions between perceived discrimination and neighborhood resources in relation to child social communication at age 2. All participants with complete or partial data were included in analyses using full information maximum likelihood estimation with robust standard errors (Enders & Bandalos, 2001).

## Results

Table 1 presents descriptive statistics for study variables. Table S5 presents bivariate correlations between study variables.

**Table 1:** Descriptive statistics for all study variables

	*Time*	*N*	*M*	*SD*	*Min*	*Max*		*Time*	*N*	*%*
Neighborhood SES	1	251	.000	1.00	−2.20	2.70	*Household Income*	3		
COI Education Domain	1	251	.000	1.00	-.90	2.31	Less than $20,000		25	10.0
COI Health & Environment Domain	1	251	.000	1.00	−1.24	2.54	$20,000 to $60,000		45	17.9
COI Social & Economic Domain	1	251	.000	1.00	-.99	2.00	$60,000 to $100,000		37	14.7
Gestational Age (in weeks)	2	251	38.78	1.53	29	41	More than $100,000		115	45.8
*Maternal Age*	2	251	32.28	5.23	18	46	Postpartum Depression/GAD	3	48	19.1
Impaired Maternal Bonding	3	218	17.74	5.27	12	48	*Maternal Minoritized Race Status*	5	122	48.6
Maternal Perceived Discrimination	3	214	15.92	8.22	10	49	*Parity: Prior Children vs. no Prior Children*	2	130	51.8
Observed Social Engagement	4	144	.000	1.00	−2.53	2.19	*Participant Child Sex: Female*	2	126	50.2
*Observed Sensitive Parenting*	4	152	.005	.91	−2.18	2.38				
*Child age (in months)*	4	163	12.95	.95	12	15				
CBCL SC Items (mother report)	5	236	.69	1.53	0	13				
ASQ SC Items (mother report)	5	251	1.82	2.63	0	20				
CBCL SC Items (2nd caregiver report)	5	210	.80	.188	0	12				
ASQ SC Items (2nd caregiver report)	5	212	1.91	2.65	0	16				
Observed Social Engagement	5	241	.000	1.00	−1.79	2.79				
Observed Sensitive Parenting	5	239	.000	1.00	−2.95	2.69				
Language Production	5	240	.000	1.00	−1.42	5.41				
*Child age (in months)*	5	251	26.43	.95	24	29				

Data were collected in pregnancy (time 1), medical record data on childbirth (time 2), mother-report data collected postpartum (time 3), observational data at age 1 (time 4), and mother-report, secondary caregiver-report, and observer ratings at age 2 (time 5). Covariates are italicized. ASQ = Ages and Stages Questionnaire; CBCL = Child Behavior Checklist; COI = Child Opportunity Index; SC = Social Communication; SES = Socioeconomic Status

### Multi-Method Latent Factor of Social Communication Difficulties at Age 2

The multi-method social communication difficulties factor showed excellent model fit (χ2(1) = 1.04; CFI = 0.999; TLI = 0.996; RMSEA = 0.01; Figures S3-4). Standardized factor loadings ranged from 0.34 to 0.63. Evidencing convergent validity, social engagement difficulties at age 1 related to more social communication difficulties at age 2 (*β* = 0.33, *p* < 0.05, Table S6). Likewise, social communication difficulties at age 2 were related to greater early child depression (*β* = 0.67, *p* < 0.001), anxiety (*β* = 0.36, *p* < 0.001), ADHD (*β* = 0.40, *p* < 0.001), and ODD (*β* = 0.29, *p* < 0.01) symptoms based on mother reports on DSM-5 oriented scales from the CBCL (see Supplemental Materials and Table S7). Higher levels of observed sensitive parenting at age 2 were also related to fewer child social communication difficulties (*β* = −0.84, *p* < 0.001; Table S8).

### Risk Pathways to Social Communication Difficulties in Toddlerhood

**Fig. 2:**
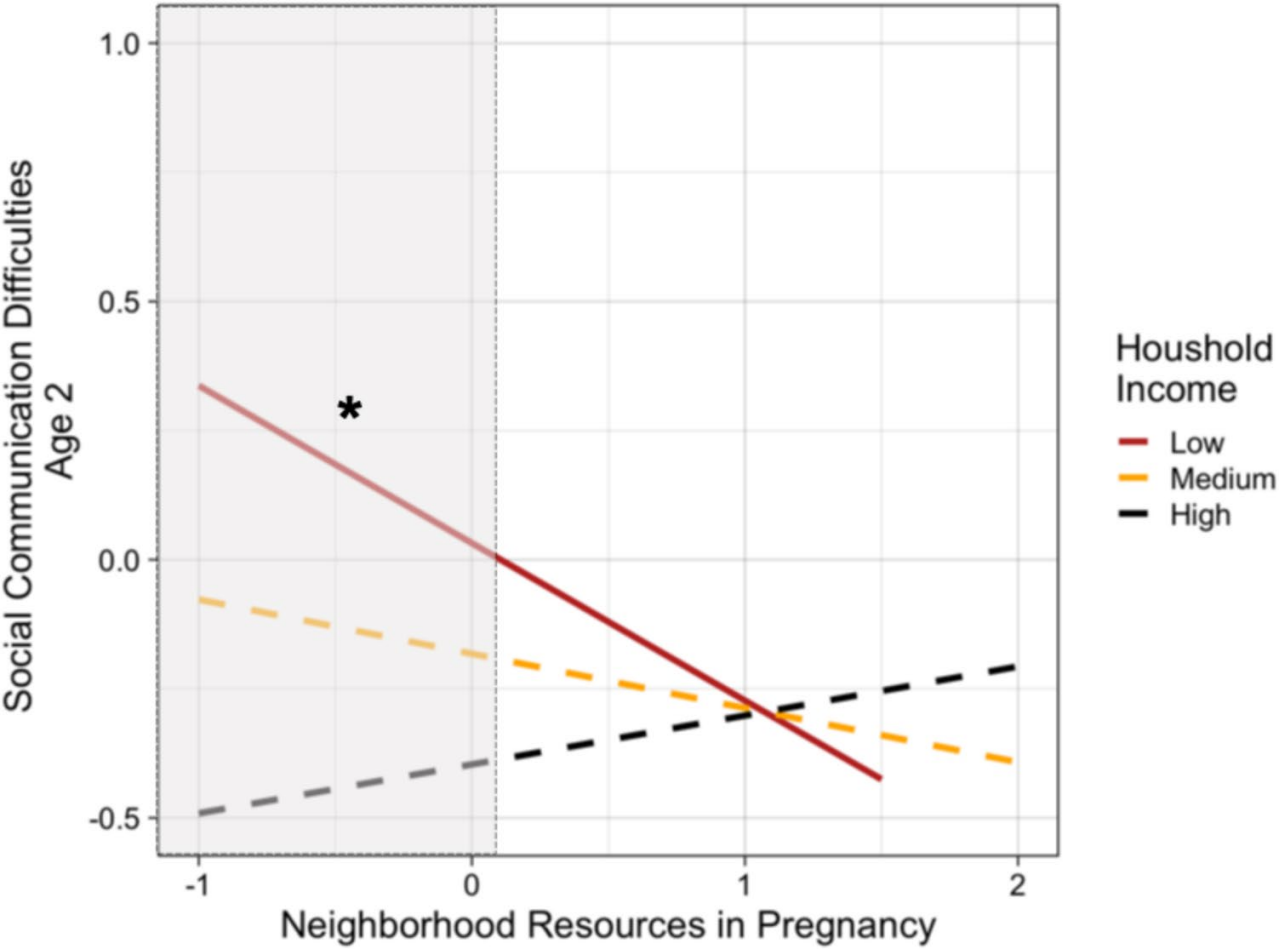
Low neighborhood resources in pregnancy were related to more child social communication difficulties at age 2 only for families with low household incomes during the postpartum period. *Note*. Simple slopes were plotted at mean levels, 1 SD above the mean, and 1 SD below the mean for household income. Fewer neighborhood resources were related to increased social communication difficulties at low (B = -.33, SE = .14, *p* < .05), but not medium (B = -.14, SE = .10, *p* = .17) or high (B = .05, SE = .11, *p* = .65) levels of household income. Region of significance (*p < .05) is shown in grey shading (i.e., at centered values of neighborhood resources less than .01)

For child influences, lower gestational age at birth related to more social communication difficulties at age 2 (*β* = −0.19, *p* < 0.05, Table 2, Figure S4). For family influences, impaired postpartum bonding (*β* = 0.27, *p* < 0.05) and low income (*β* = −0.39, *p* < 0.01) were also related to more social communication difficulties at age 2 (Table 2, Figure S4). Neither maternal mental health (*β* = −0.11, *p* = 0.29) nor neighborhood resources during pregnancy (*β* = −0.10, *p* = 0.29) were directly related to social communication difficulties at age 2. However, there was an interaction between pregnancy neighborhood resources and household income in relation to social communication difficulties at age 2 (*β* = 0.20, *p* < 0.05; Table 2). Fewer neighborhood resources related to more child social communication difficulties for families with low household incomes (*B* = −0.33, *SE* = 0.14, *p* < 0.05), but not those with mean (*B* = −0.14, *SE* = 0.10, *p* = 0.17) or high (*B* = 0.05, *SE* = 0.11, *p* = 0.65) incomes (Fig. 2). In post hoc analyses, we replicated findings controlling for mother-reported pandemic worries during pregnancy (Table S9) and observed social communication at age 1 (Table S10). Controlling for observed sensitive parenting at age 2, we replicated the main effect of family income (*β* = 0.20, *p* < 0.05), but not impaired maternal bonding (*β* = −0.01, *p* = 0.93) (Table S11).

**Table 2:** Results of path models examining main and interactive effects of peripartum risk factors on latent child social communication difficulty scores at age 2

	Main Effects			Neighborhood Level Interactions		
	*B*	*SE*	*β*	*B*	*SE*	*β*
*Covariates*						
*Child Age*	-.08	.14	-.06	-.11	.15	-.08
*Child Sex*	.30	.24	.12	.29	.24	.12
*Parity*	.05	.29	.02	-.07	.31	-.03
*Maternal Minoritized Status*	.26	.46	.10	.36	.50	.14
*Maternal Age (time 2)*	.01	.02	.06	.02	.03	.10
*Main Effects*						
Gestational Age	**-.16**	**.07**	-.19*	**-.18**	**.07**	-.22**
Postpartum Income	**-.44**	**.17**	-.39**	-.31	.19	-.27
Maternal Mental Health	-.32	.31	-.11	-.34	.32	-.11
Impaired Maternal Bonding	**.07**	**.03**	.27*	**.06**	**.02**	.25*
Neighborhood Resources (NR)	-.12	.11	-.10	-.21	.13	-.16
*Interactions*						
NR x Gestational Age				.07	.07	.08
NR x Postpartum Income				**.29**	**.14**	.20*
NR x Maternal Mental Health				-.22	.38	-.08
NR x Impaired Maternal Bonding				-.02	.03	-.07

We modeled pathways from all covariates (child age, child sex, parity, maternal minoritized status, and maternal age at the birth of the target child [time 2]) to all other predictors in the models. Covariances were allowed between predictors. *p < .05, **p < .01

**Fig. 3:**
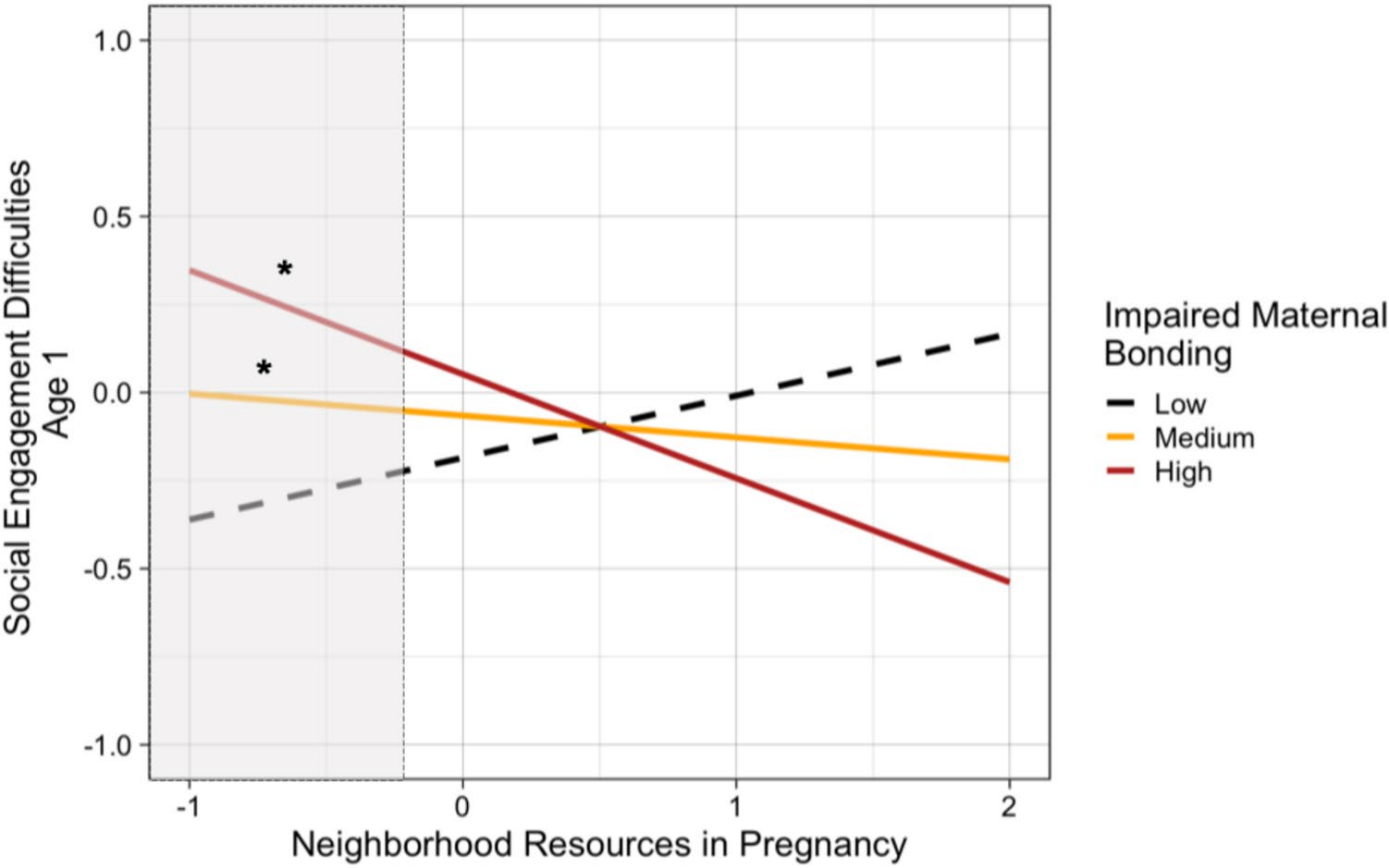
Low neighborhood resources in pregnancy were related to more observed social engagement difficulties at age 1 specifically when there was impaired postpartum maternal bonding. *Note*. Simple slopes were plotted at mean levels, 1 *SD* above the mean, and 1 *SD* below the mean for postpartum impaired maternal bonding. Lower neighborhood resources related to more social engagement difficulties at high (*B* = -.27, *SE* = .12, *p* < .05) and medium (*B* = -.03 *SE* = .02, *p* < .05) levels of impaired maternal bonding, but not at low levels (*B* = .20, *SE* = .12, *p* = .08). Region of significance (**p < .01) is shown to the left of the dotted line (i.e., at centered values of neighborhood resources less than -.32)

We partially replicated findings from age 2 using data from the subsample of families with data at age 1. There was an interaction between neighborhood resources in pregnancy and maternal impaired bonding during the postpartum period in relation to child social engagement difficulties at age 1 (*β* = −0.23, *p* < 0.05, Table S12), such that lower neighborhood resources related to more child social engagement difficulties for dyads with high (*B* = −0.27, *SE* = 0.12, *p* < 0.51) or mean (*B* = −0.03, *SE* = 0.02, *p* < 0.05), but not low (*B* = 0.20, *SE* = 0.12, *p* = 0.08) levels of impaired bonding (Fig. 3). Findings were similar controlling mother-reported pandemic worries in pregnancy (Table S13) and observed sensitive parenting at age 1 (*β* = −0.16, *p* = 0.05) (Table S14).

Supplementary analyses did not reveal significant main (*β* = −0.15, *p* = 0.16) or interactive (*β* = 0.0, *p* = 0.89) effects of postpartum maternal perceived discrimination on child social communication difficulties at age 2 (Table S15).

## Discussion

We leveraged multi-method assessments across pregnancy and the first two years of life to characterize risk factors for child social communication difficulties. Drawing on Bronfenbrenner’s ecological systems model of development (Bronfenbrenner, 1992), we investigated child (i.e., gestational age), family (i.e., household income, maternal postpartum depression or anxiety, maternal bonding), and neighborhood (i.e., neighborhood resources) factors. Consistent with prior research (van Noort-van der Spek et al., 2012), earlier gestational age at birth was associated with more social communication difficulties at age 2. Although the effect of gestational age was not qualified by interaction with neighborhood resources, it is worth noting that few of the children included in the present study were born preterm (i.e., gestational age < 37 weeks; *n* = 16, 6.4%). Thus, effects of gestational age may have been underestimated. Future work is needed to examine interactive effects of preterm birth and low neighborhood resources on social communication difficulties among larger samples of preterm children.

Consistent with prior research (Le Bas et al., 2020), impaired maternal postpartum bonding related to more child social communication difficulties at age 2. Prior research suggests that mothers who report stronger bonds with their infant experience less parenting stress (Escribano et al., 2022) and implement more positive parenting strategies (Thomson et al., 2014), which have, in turn, been linked to improved child socioemotional skills (Batool & Lewis, 2020), cognition and language use (Prime et al., 2023), and effortful control (Klein et al., 2018) across childhood. Our results expand this literature by focusing on very early maternal bonding (i.e., 10–15 weeks postpartum), pointing to an important window for targeting preventative intervention efforts to improve the parent–child relationship and promote positive parenting.

Although maternal bonding was not directly related to social engagement difficulties at age 1, there was a significant interaction between neighborhood resources in pregnancy and impaired maternal bonding in the postpartum period, such that the effects of low neighborhood resources on age 1 social engagement difficulties were only significant for dyads with greater impairments in bonding. This finding is consistent with prior research documenting critical interactions between parenting and context in relation to child outcomes (Hyde et al., 2020). For example, links between neighborhood disadvantage and school violence and child behavior problems were buffered by positive parenting (Roche et al., 2007). Interestingly, fewer neighborhood resources only related to social engagement difficulties in the context of low maternal bonding. That is, better maternal bonding, characterized by mothers’ positive feelings about their child and beliefs about their ability to provide nurturing parenting (Freeman, 2017), served to buffer the influences of fewer neighborhood resources on social engagement difficulties at age 1.

Of note, after controlling for observed sensitive parenting at age 2, impaired postpartum bonding was unrelated to social communication difficulties at age 2, suggesting that concurrent parenting experiences may buffer negative early bonding impacts (or indexing some shared method variance, since observed social communication included observer ratings from the same tasks used to rate sensitive parenting). However, results suggest that early impairments in bonding may reflect more short-lived adjustment difficulties in the context of this community sample of dyads. Importantly, prior research shows that sensitive caregiving in the first three years of life is critical to establish healthy attachment relationships and positive child development (DePasquale & Gunnar, 2020). Extant research also indicates worse cognitive and social outcomes for toddlers whose mothers report chronic depression and persistently low parenting sensitivity (NICHD Early Child Care Research Network, 1999). Taken together, our findings provide support for prevention efforts aimed at increasing sensitive parenting and maternal-child bonding throughout infancy and toddlerhood.

Finally, lower household income during the postpartum period was associated with more child social communication difficulties at age 2. Again, there was an interaction with the broader context, such that low household income in concert with fewer neighborhood resources were related to more social communication difficulties at age 2. Household income continued to be related to child social communication difficulties at age 2 even controlling for sensitive parenting at age 2. Together, results are consistent with evidence that families in lower income households situated within already economically disadvantaged neighborhoods suffer the cumulative effects of financial and social stress (e.g., working multiple shifts, experiencing health problems), but without the benefits of protective neighborhood resources (e.g., access to healthy food, safe play spaces), which might otherwise promote positive child development (Acevedo-Garcia et al., 2014). Findings expand our knowledge of how living in a neighborhood with few economic and social resources can increase chronic stress within the family (Masarik & Conger, 2017). These stressors may be compounded when accompanied by early maternal bonding difficulties, leading to parents’ decreased beliefs about their ability to effectively soothe their child or meet their needs (Franco et al., 2010). Our findings are among the first to document how the interplay of early environmental influences, such as parenting and neighborhood context, shape risk for psychopathology before overt symptoms can be reliably detected (Wakschlag et al., 2019).

Together, our findings suggest that preventative interventions deployed within the peripartum period could be especially impactful if they target positive parenting and family level factors (e.g., supporting parents to cope with parental stress and improve infant bonding), particularly among families living in lower resourced neighborhoods (Masarik & Conger, 2017; Roche et al., 2007). Such efforts could adopt a tiered approach, composed of a universal primary prevention program followed by a targeted secondary prevention for families with greater needs (Canfield et al., 2023). Other efforts include cash-based interventions during infancy, such as the Baby’s First Years study, which is testing how poverty reduction can improve child development outcomes through the provision of unconditional cash transfers to families (Noble et al., 2021). Preliminary outcome data suggest that infants of families randomized to receive high-cash gifts showed differential brain activation in regions linked to cognitive functioning (Troller-Renfree et al., 2022). Alongside our findings, similar poverty reduction efforts aimed at individual families could help benefit children’s early social communication skills, including by reducing parental stress or helping parents to invest in resources that enrich a child’s early environment (Noble et al., 2021). Importantly, although we did not find an interaction between perceived discrimination and neighborhood resources, 90% of mothers who identified as Black and/or Latina/e/o/x lived in neighborhoods below the mean for the neighborhood resources factor. Future work is needed to further evaluate how living in poverty impacts the emergence of child social communication difficulties, including among minoritized families who, because of the legacy of structural racism, may be more likely to live in neighborhoods with fewer resources (Njoroge et al., 2023).

Despite many strengths (i.e., strong theoretical framework, repeated prospective assessments from pregnancy through the second year of life), findings from our study must be considered within the context of several limitations. First, findings did not fully replicate across ages 1 and 2, which could have been due to unavoidable differences in measurement approach, including shared method variance at age 2, but not age 1 (e.g., report measures at age 2 that were not collected at age 1). Second, we relied solely on an observational design, prohibiting causal claims about relationships between variables. Moreover, models did not capture inherent bidirectionality over time between maternal bonding and child social communication abilities. Thus, we did not model whether children with lower affiliative behavior evoke less social input from parents over time (Klahr et al., 2013). Randomized controlled trials targeting parent–child bonding and parenting (e.g., Family Check-Up; Shaw et al., 2019) or more sophisticated longitudinal study designs (e.g., ecological momentary assessment) (Russell & Gajos, 2020) could help disentangle these pathways. Third, we assessed a community sample with relatively low levels of clinically severe social communication problems. Results should be replicated in clinic-referred samples of children. Fourth, maternal depression/anxiety was unrelated to child social communication difficulties. Future work needs to replicate or explore similar models in treatment-seeking parents or samples with a high proportion of individuals meeting screening thresholds for postpartum depression or anxiety. Fifth, given our sample size and the number of parameters investigated, we estimated a latent neighborhood resources factor rather than examine each COI factor independently. Future research using larger samples is needed to examine finer-grained neighborhood influences. Sixth, our cohort was originally recruited during the pandemic, which had widespread consequences for child development and mental health (Binagwaho & Senga, 2021). For example, many families contended with financial instability and the full-time care and education of their children during their pregnancy and the early postpartum phase. Of note, in supplemental analyses, we replicated our findings after controlling for mother-reported pandemic worries. Future research, however, should attempt to replicate our findings among samples of children who were not born at the height of a once-in-a-generation stressful event. Finally, although we sampled for equal representation of individuals identifying their race as Black and non-Latino/a/e/x White, our sample is not representative of the US population and results may not generalize to other racial groups.

In sum, we provide new insights into the interplay between the child, family, and neighborhood risk factors in relation to child social communication difficulties, which can signal risk for various forms of psychopathology, including depression (Meagher et al., 2009), disruptive behavior (Donno et al., 2010), and autism spectrum disorder (Costescu et al., 2022). Our results emphasize the importance of implementing early preventative interventions to help support early maternal bonding within the first few months of life, along with interventions that target families living within less advantaged neighborhoods (Leventhal et al., 2006). Established preventative interventions are effective in addressing many of these pathways, including those that target parent–child bonding and parental stress (Shieh et al., 2023), financial insecurity (Noble et al., 2021), and families living within lower resourced neighborhoods (Shaw et al., 2019). Research is needed to establish whether such interventions can be adapted at scale and implemented on a widespread population level, perhaps through a tiered approach (Canfield et al., 2023) or beginning during pregnancy (Shieh et al., 2023) to best support the parent–child dyad across development.

## Supplementary Information

Below is the link to the electronic supplementary material.

Supplementary file1 (DOCX 1425 KB)

## Data Availability

The data that support the findings of this study are available from SCP and RW upon reasonable request.
