# Supplementary material for: Characterizing Social Communication Difficulties in Young Children within a Longitudinal Ecological Systems Framework
Source: Res Child Adolesc Psychopathol. 2025 Mar 24;53(4):485–97. doi: 10.1007/s10802-025-01308-y (PMC12031844; doi:10.1007/s10802-025-01308-y)
Supplement: Supplementary file 1 — Supplementary file1 (DOCX 1425 KB) [file 10802_2025_1308_MOESM1_ESM.docx]

**Supplemental Materials**

Characterizing Social Communication Difficulties in Young Children within a Longitudinal Ecological Systems Framework

Table of Contents

[Supplemental Methods 4](#_Toc191566849)

[Participants and Procedures 4](#_Toc191566850)

[Three Bags Procedure 5](#_Toc191566851)

[Observed Child Expressed Language 6](#_Toc191566852)

[Exosystem Level Risk Factors 7](#_Toc191566853)

[Child Psychopathology Symptoms 9](#_Toc191566854)

[Additional Model Covariates 9](#_Toc191566855)

[Supplemental Results 11](#_Toc191566856)

[Exploratory Factor Analysis (EFA) of Mother and Secondary Caregiver-Report Social Communication Items. 11](#_Toc191566857)

[Neighborhood Resources Latent Factor Score. 11](#_Toc191566858)

[Results using the Continuous Maternal Mental Health Scores. 11](#_Toc191566859)

[Supplemental References 13](#_Toc191566860)

[Supplemental Tables 15](#_Toc191566861)

[Table S1. Data availability across all five study time points for the current sample 16](#_Toc191566862)

[Table S2. Summary of Measures across Time Points 17](#_Toc191566863)

[Table S3. List of Parent-Report Items used in the Social Communication Multimethod, Multiformat Measure 19](#_Toc191566864)

[Table S4. Standardized factor loadings for the neighborhood resources latent factor 20](#_Toc191566865)

[Table S5. Bivariate correlations for all study variables 21](#_Toc191566866)

[Table S6. Observed social engagement difficulties at age 1 related to higher social communication difficulties factor scores at age 2 22](#_Toc191566867)

[Table S7. Cross-sectional associations between higher social communication difficulties factor scores and greater internalizing and externalizing symptoms at age 2 23](#_Toc191566868)

[Table S8. More observed sensitive parenting behaviors is related to decreased social communication difficulties at age 2 25](#_Toc191566869)

[Table S9. Results of path models examining main and interactive effects of peripartum risk factors on social communication difficulties at age 2, controlling for maternal COVID-19 related worries during pregnancy 26](#_Toc191566870)

[Table S10. Results of path models examining main and interactive effects of peripartum risk factors on child social communication difficulties at age 2, controlling for observed social engagement at age 1 27](#_Toc191566871)

[Table S11. Results of path models examining main and interactive effects of peripartum risk factors on social communication difficulties at age 2, controlling for observed sensitive parenting at age 2 28](#_Toc191566872)

[Table S12. Results of path models examining main and interactive effects of peripartum risk factors on observed social engagement difficulties at age 1 (*n*=163) 29](#_Toc191566873)

[Table S13. Results of path models examining main and interactive effects of peripartum risk factors on observed social engagement difficulties at age 1 (*n*=163), controlling for maternal COVID-19 related worries during pregnancy 30](#_Toc191566874)

[Table 14. Results of path models examining main and interactive effects of peripartum risk factors on observed social engagement difficulties at age 1 (*n*=163), controlling for observed sensitive parenting at age 1 31](#_Toc191566875)

[Table S15. Results of path models examining main and interactive effects of peripartum risk factors, including maternal perceived discrimination on social communication difficulties at age 2 32](#_Toc191566876)

[Supplemental Figures 34](#_Toc191566877)

[Figure S1. Flow diagram of participation across all five study time points 35](#_Toc191566878)

[Figure S2. Toys for observational parent-child interactions tasks 36](#_Toc191566879)

[Figure S3. Multi-method social communication difficulties latent factor 37](#_Toc191566880)

[Figure S4. Distribution of child social communication difficulty factor scores at age 2 38](#_Toc191566881)

[Figure S5. Lower gestational age, household income, and impaired maternal bonding were related to greater social communication difficulties at age 2 39](#_Toc191566882)

# **Supplemental Methods**

## **Participants and Procedures**

The current sample included *N=*251 mother-child dyads who were recruited for a sub-study from a larger longitudinal perinatal cohort (i.e., the Intergenerational Exposome (IGNITE) study). Data were collected during pregnancy (time 1), at childbirth (time 2), 10-15 weeks postpartum (time 3), child age 1 (time 4), and child age 2 (time 5) (**Figure S1**). To recruit the larger cohort, a medical record search identified 3,548 pregnant individuals ≥18 years receiving medical and prenatal care through University of Pennsylvania health system between April 17, 2020, and May 1, 2020. Individuals were invited through email to participate in an online REDCap survey for the Intergenerational Exposome (IGNITE) study. Of the 3,548 individuals initially contacted, 1,173 individuals completed the online survey at time 1 (gestational age [GA], *M*=24.6 weeks, *SD*=8.4). Time 2 data were collected from the medical record system regarding childbirth experiences for 1,105 individuals who gave birth within the University of Pennsylvania health system. At time 3, we obtained postpartum survey data from *n*=823 (76% retention; *M*=11.8 weeks postpartum, SD=4.2) (Waller et al., 2022).

When children were 2 years old, we started a new sub-study, the Prenatal to Preschool (P2P) study (R01MH128593; Njoroge et al., 2023), which recruited a subsample (*N*=251) of the original cohort who identified as White (*n*=129 51.4%) or Black or African American (*n*=122, 48.6%) (child age, *M*=26.43 months, *SD*=.95). Six individuals also self-identified as being Latino/a/e/x (2.4%). Our recruitment strategy was targeted to address the broader goals of the P2P study, focused on investigating the impact of the syndemic (the intersection of systemic racist conditions and the COVID-19 pandemic) on maternal mental health and child development outcomes (Gur et al., 2020; Njoroge et al., 2022). Data was collected using questionnaires and from an online study visit (time 5). A subset (*n=*163) of the 251 dyads also had available data from a separate online visit at age 1 (child age, *M*=12.95 months, *SD*=.95; White, *n*=82 50.3%; Black or African American, *n*=81, 49.7%). Of the *N=*251 dyads with data from the 2-year visit, 61% had data available from all five time points (*n*=153; pregnancy, delivery, postpartum, year 1, and year 2 visits), 32% had only 4 time points (*n*=81), and 7% had only 3 time points (*n*=17). Data was available from all participants at time 1, 2, and 5. As described in the analytic strategy, we followed recommendations regarding missing data using full information maximum likelihood estimation with robust standard errors (Enders & Bandalos, 2001). Thus, models addressing our study aims leveraged the full P2P study sample of *N*=251 at time 5. See **Table S1** for additional information on data availability.

## **Three Bags Procedure**

Mothers and children were video recorded during a semi-structured online visit adapted after the Three Bags Procedure (Vandell, 1979). In the original procedure caregiver-child dyads are led through two different “situations”; a free play (10 minutes) and structured play situation (20 minutes). In the structured play situation caregivers were told that they should spend some time playing with their child with four different toys; crayons and paper, picture book, cash register with pennies, and a “surprise box” (Vandell, 1979). In the present study mother-child dyads were led through four different situations, each lasting two minutes: free play (parent and child played freely with a dinosaur toy), a structured task (child plays with a cube shape puzzle, parent is instructed to provide the child with any assistance they think is needed), and a storybook reading task (parents were asked to read a wordless book with their child) (**Figure S1**).

We assessed social communication difficulties based on observer ratings of child social engagement during this procedure. The child social engagement code assessed the extent to which the child (a) showed, initiated, and/or maintained interaction with the parent and (b) communicated positive regard and/or positive affect to the parent. At the higher end of the scale, the child expressed sustained positive affect toward the parent (e.g., big smiles, laughter, parent-directed vocalizations) and frequently attempted to interact with the parent. A team of trained researchers coded the interactions, with coders required to code 10 or more videos to establish inter-rater reliability before coding independently. Weekly meetings were held by the coding team to maintain fidelity. Coders watched each task three times before rating child social engagement from 1 (very low) to 7 (very high). Inter-rater reliability was calculated on a random 15% of videotapes. Inter-rater reliability for the child engagement construct was high across tasks (*range,* ICC=.76-.91). Scores across tasks were moderately to highly correlated (range, *r=*.51-.73, *p*<.001) and were combined into a single observed measure to reflect child social engagement across contexts. To index difficulties, we re-coded scores such that higher ratings indexed lower social engagement (i.e., greater social communication difficulty).

## **Observed** **Child Expressed Language**

We derived an objective metric of social communication based on child expressed language during the age 2 study visit. As described above, parent-child dyads completed the 3 tasks adapted from the Three Bags procedure. Dyads also completed a fourth clean-up task (parents and children played with a farmer’s market basket toy and then had to unexpectedly return all items to the basket; Figure S1). To derive a metric of child language expression, a team of three trained researchers transcribed child verbal expressions from recorded interactions for all four tasks of the online visit. All transcription data was uploaded into the 2022 updated version of Linguistic Inquiry and Word Count (LIWC) Processing software program (McDonnell et al., 2020). A transcription manual was followed to maintain consistency. For our analysis, we used the total child word count divided by the length of the parent-child interaction tasks (length [minutes], M=14.88, SD=2.93, range=8-41). A second trained researcher transcribed a random 20% of the videos, with high inter-rater reliability between the transcriptions (ICC=.85).

Of note, we also examined the following LWIC categories; affiliation (e.g., “we”, “help”, “us”), affect (e.g., emotion words, positive and negative tone words such as “good” and “bad”) and social process (e.g., “help”, “please”, “you”, “we”) based-words. Inter-rater reliability was high for the affiliation (ICC=.95) and social process (ICC=.95) categories, but low for affect words (ICC=.67). Thus, in addition to total word expression, we examined the affiliation and social process categories in future analyses. However, due to low variability and incident rate across the affiliation (*M*=3.14, *Std.*=4.76) and social process (*M*=5.10, *Std.*=5.64) categories, both of these indicators did not load significantly onto the child social communication factor. As such we utilized overall child expressed language in future analyses.

## **Exosystem Level Risk Factors**

**Calculation of Child Opportunity Index (COI) Scores (time 1)**. The COI captures child neighborhood opportunity by focusing on a broad range of neighborhood level factors including; neighborhood social structure and economic resources (e.g., neighborhood poverty and employment), environmental quality (e.g., air pollution), and resources for healthy living (e.g., green space, healthy food outlets, walkability). We obtained COI 2.0 overall z-scores for 2015 from the index.csv data file on [data.diversitydatakids.org](http://data.diversitydatakids.org/). Of note, the COI 2.0 ZIP code data are ZIP code level estimates derived from 2015 COI 2.0 census tract data. We then merged this current dataset based on ZIP code. For more information of how the COI 2.0 ZIP code estimates were derived see <https://data.diversitydatakids.org/dataset/coi20_zipcodes-child-opportunity-index-2-0-zip-code-data>. In the present study nationally-normed data was used.

Overall, child opportunity levels were ranked at “Very Low” for 49.6% (*n*=118), “Low” for 19.7% (*n*=47), “Moderate” for 5% (*n*=12), “High” for 10.9% (*n*=26), and “Very High” for 14.7% (*n*=35) of the sample.

***Neighborhood Resources Factor (time 1).*** We derived a latent factor representing neighborhood resources that combined data from four geocoding-based assessments of parent’s ZIP code during pregnancy. First, we included a composite neighborhood socioeconomic status (SES) factor derived from census-based geocoding of neighborhood-level variables (e.g., percent in poverty, percent married, and median family income) (Moore et al., 2016). Second, we included the three composites of the COI: neighborhood social structure and economic resources (e.g., neighborhood poverty and employment), environmental quality (e.g., air pollution) and resources for healthy living (e.g., green space, healthy food outlets, walkability), and educational resources (e.g., early childhood education centers, school poverty). We conducted confirmatory factor analysis in Mplus version 8 (Muthén & Muthén, 1998) specifying all four indicators to load onto an overarching latent factor representing neighborhood resources (**Table S5**). Factor scores were then extracted and used as an observed score in subsequent main and interactive effects model in order to aid in parsimony and ease of interpretation.

## **Child Psychopathology Symptoms**

**Mother-Reported Child Psychopathology (time 5).** In supplemental analyses we regressed mother-reports of child psychopathology symptoms at age 2 onto our multi-method social communication difficulties factor. More specifically, when the children were 2 years old, mothers were asked to complete the preschool version of the Child Behavior Checklist (CBCL; Achenbach & Rescorla, 2000). We utilized four of the DSM-5 oriented scales to index child depression (10 items), anxiety (10 items), attention-deficit/hyperactivity disorder (ADHD; 6 items), and oppositional defiant disorder (ODD; 6 items) symptoms. Items were rated on a 3-point Likert scale (0= rarely or never, 1=sometimes, 2=often or always). Items were summed to form a total score (*Depression*, N=242, *M*=0.92, Std=1.52, α=.63; *Anxiety*, N=241, *M*=1.67, Std=1.65, α=.47; *ADHD*, N=243, *M*=3.02, Std=2.38, α=.73; *ODD*, N=239, *M*=1.51, Std=1.87, α=.78). See **Table S7** for results.

## **Additional Model Covariates**

**Mother-Reported Pandemic Worries (time 1).**  In supplemental analyses we covaried for mother-reported pandemic worries assessed during pregnancy. In the pregnancy survey mothers reported their level of concerns for six general worries about the COVID-19 pandemic (e.g., “dying from COVID-19” or “family members getting COVID-19”). Items were rated on a 1-to-5-point scale (e.g., 1=“Not at all”; 3=“A moderate amount”; 5=“A great deal”). Items were summed to form a total score (N=251, *M*=16.56, Std=5.75, α=.86). See **Tables S8** and **S11** for results controlling for COVID-19 worries at age 1 and 2 respectively.

**Observed Sensitive Parenting (time 4 and 5)**. In supplemental analyses we covaried for observed sensitive parenting behaviors at age 1 and 2 respectively. In addition to coding for child social engagement during the Three Bags procedure we also coded for sensitive and positive parenting behaviors at age 1 and 2 using the Parent Child Interactions Rating System (PCIRS). Inter-rater reliability was calculated on a random 20% of videotapes stratified across coders (age 1, ICC range=.72-91; age 2, ICC range=X-X). Three subscales of parenting behaviors were assessed; 1) Sensitive parenting, which captured attunement to the needs and interests of children and effective monitoring and responding to their behavior; 2) Positive Regard, which captured positive feelings directed toward the child; and 3) Stimulation of cognitive development, which captured facilitation of child learning through explanations and demonstrations. Ratings were made using a 7-point scale (1= very low, 7= very high), with higher ratings indicating more positive parenting. We then used factor analysis to create an overall sensitive and positive parenting factor. For more information see (Waller et al., 2024).

# **Supplemental Results**

**Exploratory Factor Analysis (EFA) of Mother and Secondary Caregiver-Report Social Communication Items.** Separately for the ASQ and CBCL items we conducted an EFA, first examining mother-reported items and then secondary caregiver-reported items. For mother-report on the ASQ items, factor analysis revealed superiority of a unidimensional solution (**Table S4**). Eigenvalues greater than 2.00 emerged only for the first factor (F1, λ =3.81; F2, λ =1.02; F3, λ =1.01; F4, λ =.91; F5, λ =.80). Similarly, for mother-report on the CBCL items, factor analysis revealed unidimensional solution (**Table S4**). Further, eigenvalues greater than 2.00 emerged only for the first factor (F1, λ =3.54; F2, λ =1.09; F3, λ =.83; F4, λ =.64; F5, λ =.47).

Regarding the secondary caregiver-report on the ASQ items, factor analysis revealed superiority of a unidimensional solution (**Table S4**). Eigenvalues greater than 2.00 emerged only for the first factor (F1, λ =4.19; F2, λ =1.05; F3, λ =.83; F4, λ =.78; F5, λ =.71). Finally, factor analysis revealed superiority of a unidimensional solution for secondary caregiver-reported CBCL items (**Table S4**). Eigenvalues greater than 2.00 emerged only for the first factor (F1, λ =3.81; F2, λ =.97; F3, λ =.85; F4, λ =.53; F5, λ =.40).

**Neighborhood Resources Latent Factor Score.** The neighborhood resources factor showed excellent fit (χ^2^(2)= 4.36; CFI = .996; TLI = .987; RMSEA = .07). See **Table S5** for standardized factor loadings. The extracted factor scores had a mean of 0 and a SD of 1.

**Results using the Continuous Maternal Mental Health Scores.** First, bivariate correlations between continuous postpartum depression symptoms and child social communication difficulties revealed non-significant correlations at ages 1 (*r*=-.01, *p*=.87) and 2 (*r*=-.05, *p*=.50) respectively. Results were similar for continuous postpartum anxiety symptoms at ages 1 (*r*=-.05, *p*=.58) and 2 (*r*=.02, *p*=.79).

|  | **Postpartum Period** | | |
| --- | --- | --- | --- |
|  | Maternal Bonding | Maternal Depression | Maternal Anxiety |
| SED at age 1 | .15 | -.01 | -.05 |
| SCD at age 2 | .19^**^ | -.05 | .02 |
| Maternal Bonding | -- | .40^***^ | .34^**^ |
| Maternal Depression | .40^***^ | -- | .83^***^ |
| Maternal Anxiety | .34^***^ | .83^***^ | -- |

*Note*. SCD= Social Communication Difficulties; SED=Social Engagement Difficulties. ***p*<.01, ****p*<.001

Multiple regression analyses at age 1 also revealed a non-significant main effect of depression symptoms on observed social engagement difficulties (β=-.07, *p*=.42) and a non-significant interaction between depression symptoms and neighborhood resources (β=.04, *p*=.65). Results were similar for maternal anxiety symptoms (main effect, β=-.12, *p*=.14; interactive effect, β=.08, *p*=.40).

At age 2, however, there was evidence of *negative confounding*, such that when mother-reports of both postpartum depression symptoms and impaired bonding were included within the same model there was a significant main effect of *both* continuous predictors (but not when they were examined separately). That is, *fewer* depression symptoms (β=-.24, *p*<.05) and greater impaired maternal bonding (β=.34, *p*<.01) were both associated with more child social communication difficulties at age 2. In contrast, when maternal-reports of impaired bonding were dropped from the model, depression symptoms no longer had a significant main effect on social communication difficulties (β=-.12, *p*=.19). When depression symptoms were dropped from the model, the effect of impaired maternal bonding was still associated with greater child social communication difficulties, although the effect was trending (β=.23, *p*=.06).

When examining maternal anxiety symptoms at age 2 multiple regression analyses revealed non-significant main and interactive effects of symptoms on social communication difficulties (main effect, β=-.13, *p*=.12; interactive effect, β=.01, *p*=.94). Within this model a significant main effect impaired maternal bonding on child social communication difficulties still emerged (β=.29, *p*<.05).

# **Supplemental References**

Achenbach, T. M., & Rescorla, L. A. (2000). *Manual for the ASEBA preschool forms and profiles* (Vol. 30). University of Vermont, Research center for children, youth, & families.

Enders, C. K., & Bandalos, D. L. (2001). The Relative Performance of Full Information Maximum Likelihood Estimation for Missing Data in Structural Equation Models. *Structural Equation Modeling: A Multidisciplinary Journal*, *8*(3), 430–457. https://doi.org/10.1207/S15328007SEM0803_5

Gur, R. E., White, L. K., Waller, R., Barzilay, R., Moore, T. M., Kornfield, S., Njoroge, W. F. M., Duncan, A. F., Chaiyachati, B. H., Parish-Morris, J., Maayan, L., Himes, M. M., Laney, N., Simonette, K., Riis, V., & Elovitz, M. A. (2020). The Disproportionate Burden of the COVID-19 Pandemic Among Pregnant Black Women. *Psychiatry Research*, *293*, 113475. https://doi.org/10.1016/j.psychres.2020.113475

Moore, T. M., Martin, I. K., Gur, O. M., Jackson, C. T., Scott, J. C., Calkins, M. E., Ruparel, K., Port, A. M., Nivar, I., Krinsky, H. D., Gur, R. E., & Gur, R. C. (2016). Characterizing social environment’s association with neurocognition using census and crime data linked to the Philadelphia Neurodevelopmental Cohort. *Psychological Medicine*, *46*(3), 599–610. https://doi.org/10.1017/S0033291715002111

Muthén, L. K., & Muthén, B. O. (1998). *Mplus User’s Guide.* (7th ed.). Muthén & Muthén.

Njoroge, W. F. M., Tieu, T., Eckardt, D., Himes, M., Alexandre, C., Hall, W., Wisniewski, K., Popoola, A., Holloway, K., Rodriguez, Y., Kornfield, S., Momplaisir, F., Wang, X., Gur, R., & Waller, R. (2023). The impact of the pandemic on mothers and children, with a focus on syndemic effects on black families: The “Prenatal to Preschool” study protocol. *Frontiers in Psychiatry*, *14*, 1281666. https://doi.org/10.3389/fpsyt.2023.1281666

Njoroge, W. F. M., White, L. K., Waller, R., Forkpa, M., Himes, M. M., Morgan, K., Seidlitz, J., Chaiyachati, B. H., Barzilay, R., Kornfield, S. L., Parish-Morris, J., Rodriguez, Y., Riis, V., Burris, H. H., Elovitz, M. A., & Gur, R. E. (2022). Association of COVID-19 and Endemic Systemic Racism With Postpartum Anxiety and Depression Among Black Birthing Individuals. *JAMA Psychiatry*, *79*(6), 600–609. https://doi.org/10.1001/jamapsychiatry.2022.0597

Waller, R., Kornfield, S. L., White, L. K., Chaiyachati, B. H., Barzilay, R., Njoroge, W., Parish-Morris, J., Duncan, A., Himes, M. M., Rodriguez, Y., Seidlitz, J., Riis, V., Burris, H. H., Gur, R. E., & Elovitz, M. A. (2022). Clinician-reported childbirth outcomes, patient-reported childbirth trauma, and risk for postpartum depression. *Archives of Women’s Mental Health*, *25*(5), 985–993. https://doi.org/10.1007/s00737-022-01263-3

Waller, R., Paz, Y., Himes, M. M., White, L. K., Rodriguez, Y., Gorgone, A., Luby, J., Gerstein, E. D., Brady, R. G., Chaiyachati, B. H., Duncan, A., Barzilay, R., Kornfield, S. L., Burris, H. H., Seidlitz, J., Parish-Morris, J., Laney, N., Gur, R. E., & Njoroge, W. F. M. (2024). Observations of Positive Parenting from Online Parent-Child Interactions at Age 1. *Parenting, Science and Practice*, *24*(1), 39–65. https://doi.org/10.1080/15295192.2023.2286454

# **Supplemental Tables**

## **Table S1.** Data availability across all five study time points for the current sample

|  | **Time 1**  (*pregnancy)* | **Time 2**  (*childbirth)* | **Time 3**  (*10-15 weeks postpartum)* | **Time 4**  (*12 months postpartum)* |
| --- | --- | --- | --- | --- |
| **Current Sample**  **(*N*= 251)** | 251 (100%) | 251 (100%) | 224 (89.2%) | 163 (64.9%) |

*Note*. Of note, 61% had fully available data at all 5 time points (*n*=153), 32% had 4 time points (*n*=81) and 7% with 3 time points (*n*=17). Regarding missing data at Time 3, mothers who held minoritized racial identities were more likely to have missing data than mother’s who identified as white across all three of our main study variables at this time point (e.g., family income, *X*^2^=36.11, *p*<.001; maternal mental health, *X*^2^=36.11, *p*<.001; impaired maternal bonding, *X*^2^=32.73, *p*<.001). Younger mothers were also significantly more likely to have missing data on our income (*F*=5.13, *p*<.001), maternal mental health (*F*=5.13, *p*<.001), and maternal bonding (*F*=6.75, *p*<.01) measures. Dyads with older children at the age 2 visit were more likely to have missing data on our income (*F*=1.12, *p*<.05) and maternal mental health (*F*=1.12, *p*<.05) measures. There was no significant difference on the proportion of missing data based on parity or child sex. Regarding missing data at Time 4, there was no significant difference on the proportion of missing data for our observed social engagement measure based on maternal minoritized racial status, parity, child sex, maternal age, or child age.

## **Table S2.** Summary of Measures across Time Points

|  | **Demographic Covariates** | **Peripartum Risk Factors** | | | **Child Developmental Outcomes** |
| --- | --- | --- | --- | --- | --- |
|  |  | *Neighborhood Level* | *Family Level* | *Child Level* |  |
| **Time 1**  (*pregnancy*; self-report survey) |  | SES by Zip Code  COI by Zip Code |  |  |  |
| **Time 2**  (*childbirth*; self-report survey and collected from electronic health records) | Parity  Maternal Age |  |  | Gestational Age |  |
| **Time 3**  (*10-15 weeks postpartum*; self-report survey) |  |  | Postpartum Depression (EPDS)  Maternal Generalized Anxiety (GAD-7)  Impaired Maternal Bonding (PBQ)  Maternal Perceived Discrimination (EDS)  Household Income |  |  |
| **Time 4**  (*1 year postpartum*; parent-report survey and virtual laboratory visit) | Child Age |  |  |  | Social Engagement Difficulties (observed) |
| **Time 5**  (*2 years postpartum*; parent-report survey and virtual laboratory visit) | Child Age  Child Sex  Maternal Race |  |  |  | Social Communication Difficulties (mother-report, alternate caregiver-report )  Social Engagement Difficulties (observed)  Language Use (observed) |

*Note*. COI=Child Opportunity Index; EDS= Everyday Discrimination Scale; EPDS= Edinburgh Postnatal Depression Scale; GAD-7= Generalized anxiety disorder 7-item scale; PBQ=Postpartum Bonding Questionnaire; SES= Socioeconomic status.

## **Table S3.** List of Parent-Report Items used in the Social Communication Multimethod, Multiformat Measure

| **Measure** | **Item** |
| --- | --- |
| **ASQ** | Does your child look at you when you talk to him? |
|  | Does your child laugh or smile when you play with her? |
|  | Does your child greet or say hello to familiar adults? |
|  | Does your child like to be hugged or cuddled? |
|  | Is your child interested in things around them, such as people, toys, and foods? |
|  | When you point at something, does your child look in the direction you are pointing? |
|  | Does your child let you know how they are feeling with words or gestures? For example, do they let you know when they are hungry, hurt, or tired? |
|  | Does your child like to hear stories or sing songs? |
|  | Does your child like to be around other children? For example, do they move close to or look at other children? |
|  | Does your child respond to their name when you call them? For example, do they turn their head and look at you? |
| **CBCL** | Avoids looking others in the eye |
|  | Doesn't answer when people talk to him/her |
|  | Doesn't get along with other children |
|  | Seems unresponsive to affection |
|  | Shows little affection toward people |
|  | Speech problem |
|  | Withdrawn, doesn't get involved with others |

*Note*. ASQ= Ages and Stages Questionnaire; CBCL= Child Behavioral Checklist

## **Table S4**. Standardized factor loadings for the neighborhood resources latent factor

|  | *Factor Loading* | *S.E.* |
| --- | --- | --- |
| Neighborhood SES | .897*** | .02 |
| COI Education Domain | .918*** | .01 |
| COI Health & Environment Domain | .861*** | .02 |
| COI Social & Economic Domain | .999*** | .004 |

*Note*. Factor scores were then extracted and used as an observed score in subsequent main and interactive effects model in order to aid in parsimony and ease of interpretation.

COI= Child Opportunity Index; SES= Socioeconomic status.

****p*<.001

## **Table S5.** Bivariate correlations for all study variables

|  | 1 | 2 | 3 | 4 | 5 | 6 | 7 | 8 | 9 | 10 | 11 | 12 | 13 | 14 | 15 | 16 |
| --- | --- | --- | --- | --- | --- | --- | --- | --- | --- | --- | --- | --- | --- | --- | --- | --- |
| 1. Neighborhood Resources |  |  |  |  |  |  |  |  |  |  |  |  |  |  |  |  |
| 2. Gestational Age | .06 |  |  |  |  |  |  |  |  |  |  |  |  |  |  |  |
| 3. Maternal Age (time 2) | .24*** | -.11 |  |  |  |  |  |  |  |  |  |  |  |  |  |  |
| 4. Household Income | .52*** | .07 | .37*** |  |  |  |  |  |  |  |  |  |  |  |  |  |
| 5. Maternal Mental Health | -.02 | -.10 | -.08 | -.13 |  |  |  |  |  |  |  |  |  |  |  |  |
| 6. Maternal Bonding | .07 | .05 | .02 | -.05 | -.32*** |  |  |  |  |  |  |  |  |  |  |  |
| 7. Observed SED (time 4) | .07 | .05 | .003 | .18* | -.05 | -.15 |  |  |  |  |  |  |  |  |  |  |
| 8. Child Age (time 4) | -.23** | -.12 | .04 | -.03 | .01 | .10 | -.04 |  |  |  |  |  |  |  |  |  |
| 9. ASQ (MR) | -.22*** | -.12 | -.06 | -.30*** | .10 | -.19** | .14 | .09 |  |  |  |  |  |  |  |  |
| 10. CBCL (MR) | -.24*** | -.10 | -.03 | -.27*** | .06 | -.15* | .10 | .07 | .74*** |  |  |  |  |  |  |  |
| 11. ASQ (SCR) | -.20** | -.22*** | -.12 | -.34*** | .13 | -.21** | .07 | .12 | .48*** | .28*** |  |  |  |  |  |  |
| 12. CBCL (SCR) | -.14* | -.32*** | -.10 | -.31*** | .13 | -.19** | -.02 | .05 | .53*** | .40*** | .72*** |  |  |  |  |  |
| 13. Observed SED (time 5) | -.11 | -.10 | -.02 | -.09 | -.02 | -.002 | .17 | .22** | .39*** | .24*** | .17* | .10 |  |  |  |  |
| 14. Language Production | -.23*** | -.06 | -.11 | -.24*** | -.06 | -.04 | .003 | -.07 | .23*** | .24*** | .17* | .15* | .27*** |  |  |  |
| 15. Child Age (time 5) | -.12 | .01 | .03 | -.10 | -.04 | .13 | .03 | .26*** | .02 | .10 | .04 | .02 | -.10 | -.07 |  |  |
| 16. Child Sex | .05 | .04 | -.09 | -.01 | .02 | -.03 | -.01 | -.08 | .04 | .05 | -.04 | .01 | .10 | .10 | -.05 |  |
| 17. Minoritized Status | -.59*** | -.08 | -.36*** | -.70*** | .10 | .12 | -.12 | .22** | .19** | .18** | .23*** | .18** | .08 | .28*** | .18** | -.04 |

Note. ASQ= Ages and Stages Questionnaire; CBCL= Child Behavioral Checklist; MR=Mother-Report; SCR= Father/Secondary-Caregiver Report; SCD= Social Communication Difficulties; SED=Social Engagement Difficulties. **p*<.05, ***p*<.01, ****p*<.001

## **Table S6.** Observed social engagement difficulties at age 1 related to higher social communication difficulties factor scores at age 2

|  | **Social Communication Difficulties (Age 2)** | | |
| --- | --- | --- | --- |
|  | **Latent Factor** | | |
|  | *B* | *SE* | *β* |
| Social Engagement Difficulties (Age 1) | **.39** | **.17** | **.33*** |
| ***Covariates*** |  |  |  |
| *Child Age* | -.16 | .10 | -.13 |
| *Child Sex* | .38 | .20 | .16 |
| *Parity* | .23 | .30 | .10 |
| *Maternal Minoritized Racial Identity* | **.99** | **.32** | **.42***** |
| *Maternal Age* | .003 | .02 | .01 |

*Note*. We modeled pathways from all covariates to all other predictors in the models. Covariances were allowed between predictors.

**p*<.05, ***p*<.01, ****p*<.001

## **Table S7.** Cross-sectional associations between higher social communication difficulties factor scores and greater internalizing and externalizing symptoms at age 2

|  | **Social Communication Difficulties (Age 2)** | | |
| --- | --- | --- | --- |
|  | **Latent Factor** | | |
|  | *B* | *SE* | *β* |
| **Model 1: Depression Symptoms** | **.63** | **.11** | **.67***** |
| ***Covariates*** |  |  |  |
| *Child Age* | -.01 | .09 | -.01 |
| *Child Sex* | .10 | .17 | .03 |
| *Parity* | .10 | .19 | .04 |
| *Maternal Minoritized Racial Identity* | .65 | .31 | .23* |
| *Maternal Age* | .001 | .02 | .004 |
|  | *B* | *SE* | *β* |
| **Model 2: Anxiety Symptoms** | **.25** | **.08** | **.36***** |
| ***Covariates*** |  |  |  |
| *Child Age* | -.14 | .11 | -.12 |
| *Child Sex* | .24 | .21 | .10 |
| *Parity* | -.001 | .25 | .000 |
| *Maternal Minoritized Racial Identity* | .78 | .34 | .33* |
| *Maternal Age* | .01 | .02 | .03 |
|  | *B* | *SE* | *β* |
| **Model 3: ADHD Symptoms** | **.20** | **.06** | **.40***** |
| ***Covariates*** |  |  |  |
| *Child Age* | -.12 | .13 | -.09 |
| *Child Sex* | .33 | .20 | .14 |
| *Parity* | .15 | .26 | .06 |
| *Maternal Minoritized Racial Identity* | .77 | .42 | .32 |
| *Maternal Age* | .01 | .02 | .03 |
|  | *B* | *SE* | *β* |
| **Model 4: ODD Symptoms** | **.18** | **.07** | **.28**** |
| ***Covariates*** |  |  |  |
| *Child Age* | -.17 | .11 | -.14 |
| *Child Sex* | .33 | .19 | .14 |
| *Parity* | .02 | .26 | .01 |
| *Maternal Minoritized Racial Identity* | .99 | .32 | .43** |
| *Maternal Age* | .01 | .02 | .04 |

*Note*. Child symptoms of depression, anxiety, attention-deficit/hyperactivity disorder (ADHD), and oppositional defiant disorder (ODD) were derived from the DSM-5 oriented scales of the Preschool Child Behavior Checklist (CBCL; Achenbach & Rescorla, 2000).

**p*<.05, ***p*<.01, ****p*<.001

## **Table S8.** More observed sensitive parenting behaviors is related to decreased social communication difficulties at age 2

|  | **Main Effects** | | |
| --- | --- | --- | --- |
|  | *B* | *SE* | *β* |
| *Observed Sensitive Parenting* | -1.46 | .29 | -.84*** |
| ***Covariates*** |  |  |  |
| *Child Age* | -.20 | .09 | -.11* |
| *Child Sex* | .33 | .18 | .10 |
| *Parity* | -.45 | .20 | -.14* |
| *Maternal Minoritized Racial Identity* | -.27 | .19 | -.08 |
| *Maternal Age (time 2)* | .05 | .02 | .17** |

*Note*. We modeled pathways from all covariates to all other predictors. Covariances were allowed between predictors. **p*<.05, ***p*<.01, ****p*<.001

## **Table S9.** Results of path models examining main and interactive effects of peripartum risk factors on social communication difficulties at age 2, controlling for maternal COVID-19 related worries during pregnancy

|  | **Main Effects** | | | **Neighborhood Level Interactions** | | |
| --- | --- | --- | --- | --- | --- | --- |
|  | *B* | *SE* | *β* | *B* | *SE* | *β* |
| ***Covariates*** |  |  |  |  |  |  |
| *Child Age* | -.07 | .14 | -.06 | -.11 | .15 | -.08 |
| *Child Sex* | .30 | .24 | .12 | .29 | .24 | .12 |
| *Parity* | .05 | .30 | .02 | -.06 | .31 | -.02 |
| *Maternal Minoritized Racial Identity* | .26 | .46 | .11 | .37 | .50 | .15 |
| *Maternal Age (time 2)* | .01 | .02 | .06 | .02 | .03 | .10 |
| *Maternal Covid-19 Worries* | -.001 | .02 | -.01 | -.01 | .02 | -.03 |
| ***Main effects*** |  |  |  |  |  |  |
| Gestational Age | **-.16** | **.07** | **-.19*** | **-.19** | **.08** | **-.23*** |
| Postpartum Income | **-.44** | **.17** | **-.38**** | -.31 | .19 | -.27 |
| Maternal Mental Health | -.33 | .31 | -.11 | -.35 | .32 | -.11 |
| Impaired Maternal Bonding | **.07** | **.03** | **.27*** | **.06** | **.02** | **.26*** |
| Neighborhood Resources (NR) | -.12 | .11 | -.10 | -.21 | .13 | -.16 |
| ***Interactions*** |  |  |  |  |  |  |
| NR x Gestational Age |  |  |  | .07 | .07 | .08 |
| NR x Postpartum Income |  |  |  | **.29** | **.14** | **.20*** |
| NR x Maternal Mental Health |  |  |  | -.22 | .38 | -.08 |
| NR x Impaired Maternal Bonding |  |  |  | -.02 | .03 | -.07 |

*Note*. We modeled pathways from all covariates to all other predictors. Covariances were allowed between predictors. **p*<.05, ***p*<.01

## **Table S10.** Results of path models examining main and interactive effects of peripartum risk factors on child social communication difficulties at age 2, controlling for observed social engagement at age 1

|  | **Main Effects** | | | **Neighborhood Level Interactions** | | |
| --- | --- | --- | --- | --- | --- | --- |
|  | *B* | *SE* | *β* | *B* | *SE* | *β* |
| ***Covariates*** |  |  |  |  |  |  |
| *Child Age* | -.08 | .13 | -.06 | -.12 | .14 | -.08 |
| *Child Sex* | .34 | .24 | .13 | .32 | .24 | .12 |
| *Parity* | .23 | .29 | .09 | .13 | .33 | .05 |
| *Maternal Minoritized Racial Identity* | .26 | .45 | .09 | .33 | .50 | .12 |
| *Maternal Age (time 2)* | .01 | .02 | .05 | .02 | .03 | .09 |
| *Observed social communication age 1* | **.45** | **.17** | **.33**** | **.43** | **.18** | **.32**** |
| ***Main effects*** |  |  |  |  |  |  |
| Gestational Age | **-.18** | **.08** | **-.20*** | **-.20** | **.08** | **-.23**** |
| Postpartum Income | **-.51** | **.18** | **-.42**** | **-.41** | **.20** | **-.33*** |
| Maternal Mental Health | -.27 | .31 | -.08 | -.30 | .32 | -.09 |
| Impaired Maternal Bonding | **.06** | **.03** | **.22*** | **.06** | **.02** | **.21*** |
| Neighborhood Resources (NR) | -.14 | .11 | -.10 | -.19 | .13 | -.14 |
| ***Interactions*** |  |  |  |  |  |  |
| NR x Gestational Age |  |  |  | .08 | .07 | 0.08 |
| NR x Postpartum Income |  |  |  | .25 | .14 | 0.16 |
| NR x Maternal Mental Health |  |  |  | -.23 | .38 | -.08 |
| NR x Impaired Maternal Bonding |  |  |  | .001 | .03 | .002 |

*Note*. We modeled pathways from all covariates to all other predictors. Covariances were allowed between predictors. **p*<.05, ***p*<.01

## **Table S11.** Results of path models examining main and interactive effects of peripartum risk factors on social communication difficulties at age 2, controlling for observed sensitive parenting at age 2

|  | **Main Effects** | | | **Neighborhood Level Interactions** | | |
| --- | --- | --- | --- | --- | --- | --- |
|  | *B* | *SE* | *β* | *B* | *SE* | *β* |
| ***Covariates*** |  |  |  |  |  |  |
| *Child Age* | .23 | .10 | .12* | -.24 | .11 | -.13* |
| *Child Sex* | -.39 | .21 | -.11* | .39 | .22 | .11* |
| *Parity* | .62 | .25 | .17** | -.65 | .26 | -.18** |
| *Maternal Minoritized Racial Identity* | .67 | .35 | .19* | -.63 | .36 | -.17 |
| *Maternal Age (time 2)* | -.08 | .03 | -.22*** | .08 | .03 | .22** |
| *Observed Sensitive Parenting* | 1.61 | .38 | .85*** | -1.61 | .39 | -.85*** |
| ***Main effects*** |  |  |  |  |  |  |
| Gestational Age | .04 | .05 | .03 | -.05 | .06 | -0.04 |
| Postpartum Income | .34 | .20 | .20* | -.29 | .21 | -0.17 |
| Maternal Mental Health | .15 | .26 | .03 | -.14 | .28 | -0.03 |
| Impaired Maternal Bonding | -.002 | .03 | -.01 | -.001 | .03 | 0.01 |
| Neighborhood Resources (NR) | .02 | .11 | .01 | -.01 | .14 | -0.01 |
| ***Interactions*** |  |  |  |  |  |  |
| NR x Gestational Age |  |  |  | .03 | .06 | .02 |
| NR x Postpartum Income |  |  |  | .08 | .14 | .04 |
| NR x Maternal Mental Health |  |  |  | -.24 | .28 | -.002 |
| NR x Impaired Maternal Bonding |  |  |  | -.001 | .02 | -.06 |

*Note*. We modeled pathways from all covariates to all other predictors. Covariances were allowed between predictors. **p*<.05, ***p*<.01, ****p*<.001

## **Table S12.** Results of path models examining main and interactive effects of peripartum risk factors on observed social engagement difficulties at age 1 (*n*=163)

|  | **Main Effects** | | | **Neighborhood Level Interactions** | | |
| --- | --- | --- | --- | --- | --- | --- |
|  | *B* | *SE* | *β* | *B* | *SE* | *β* |
| ***Covariates*** |  |  |  |  |  |  |
| *Child Age* | .02 | .09 | .02 | .001 | .09 | .001 |
| *Child Sex* | -.04 | .17 | -.02 | -.03 | .17 | -.02 |
| *Parity* | -.33 | .18 | -.17 | -.40 | .18 | -.20* |
| *Maternal Minoritized Racial Identity* | .07 | .19 | .03 | .13 | .21 | .07 |
| *Maternal Age (time 2)* | .003 | .02 | .01 | .001 | .02 | .01 |
| ***Main effects*** |  |  |  |  |  |  |
| Gestational Age | .02 | .04 | .03 | .02 | .05 | .02 |
| Postpartum Income | .14 | .10 | .15 | .20 | .13 | .21 |
| Maternal Mental Health | -.14 | .21 | -.06 | -.09 | .20 | -.04 |
| Impaired Maternal Bonding | .03 | .02 | .17 | .03 | .02 | .13 |
| Neighborhood Resources (NR) | .02 | .10 | .02 | -.04 | .12 | -.04 |
| ***Interactions*** |  |  |  |  |  |  |
| NR x Gestational Age |  |  |  | -.01 | .06 | -.02 |
| NR x Postpartum Income |  |  |  | .10 | .13 | .09 |
| NR x Maternal Mental Health |  |  |  | -.05 | .21 | -.02 |
| NR x Impaired Maternal Bonding |  |  |  | -.05 | .02 | -.23* |

*Note*. We modeled pathways from all covariates (child age at time 4, child sex, maternal minority status, and parity) to all other predictors in the models. Covariances were allowed between predictors. **p*<.05

## **Table S13.** Results of path models examining main and interactive effects of peripartum risk factors on observed social engagement difficulties at age 1 (*n*=163), controlling for maternal COVID-19 related worries during pregnancy

|  | **Main Effects** | | | **Neighborhood Level Interactions** | | |
| --- | --- | --- | --- | --- | --- | --- |
|  | *B* | *SE* | *β* | *B* | *SE* | *β* |
| ***Covariates*** |  |  |  |  |  |  |
| *Child Age* | .02 | .09 | .02 | 0.003 | 0.09 | .003 |
| *Child Sex* | -.04 | .17 | -.02 | -0.04 | 0.17 | -.02 |
| *Parity* | -.33 | .19 | -.16 | -0.39 | 0.18 | -.19* |
| *Maternal Minoritized Racial Identity* | .07 | .19 | .04 | 0.14 | 0.21 | .07 |
| *Maternal Age (time 2)* | .002 | .02 | .01 | 0.000 | 0.02 | .001 |
| *Maternal Covid-19 Worries* | -.002 | .02 | -.01 | -0.002 | 0.02 | -.01 |
| ***Main effects*** |  |  |  |  |  |  |
| Gestational Age | .02 | .05 | .03 | 0.02 | 0.05 | .02 |
| Postpartum Income | .14 | .10 | .15 | 0.20 | 0.13 | .22 |
| Maternal Mental Health | -.14 | .22 | -.06 | -0.10 | 0.21 | -.04 |
| Impaired Maternal Bonding | .03 | .02 | .18 | 0.03 | 0.02 | .14 |
| Neighborhood Resources (NR) | .03 | .10 | .03 | -0.04 | 0.12 | -.04 |
| ***Interactions*** |  |  |  |  |  |  |
| NR x Gestational Age |  |  |  | -.01 | .06 | -.02 |
| NR x Postpartum Income |  |  |  | .10 | .12 | .09 |
| NR x Maternal Mental Health |  |  |  | -.05 | .21 | -.02 |
| NR x Impaired Maternal Bonding |  |  |  | **-.05** | **.02** | **-.23*** |

*Note*. We modeled pathways from all covariates to all other predictors in the models. Covariances were allowed between predictors. **p*<.05

## **Table 14.** Results of path models examining main and interactive effects of peripartum risk factors on observed social engagement difficulties at age 1 (*n*=163), controlling for observed sensitive parenting at age 1

|  | **Main Effects** | | | **Neighborhood Level Interactions** | | |
| --- | --- | --- | --- | --- | --- | --- |
|  | *B* | *SE* | *β* | *B* | *SE* | *β* |
| ***Covariates*** |  |  |  |  |  |  |
| *Child Age* | .01 | .08 | .01 | -.01 | .08 | -.01 |
| *Child Sex* | -.02 | .15 | -.01 | .01 | .15 | .002 |
| *Parity* | -.49 | .16 | -.24** | -.53 | .17 | -.26** |
| *Maternal Minoritized Racial Identity* | -.27 | .18 | -.13 | -.25 | .19 | -.12 |
| *Maternal Age (time 2)* | .01 | .02 | .05 | .01 | .02 | .03 |
| *Observed Sensitive Parenting* | -.57 | .08 | -.51*** | -.56 | .08 | -.51*** |
| ***Main effects*** |  |  |  |  |  |  |
| Gestational Age | .02 | .04 | .02 | .01 | .05 | .02 |
| Postpartum Income | .09 | .10 | .09 | .11 | .11 | .12 |
| Maternal Mental Health | .02 | .19 | .01 | .08 | .19 | .03 |
| Impaired Maternal Bonding | .02 | .02 | .09 | .01 | .01 | .07 |
| Neighborhood Resources (NR) | .01 | .09 | .01 | .01 | .10 | .01 |
| ***Interactions*** |  |  |  |  |  |  |
| NR x Gestational Age |  |  |  | .001 | .05 | .001 |
| NR x Postpartum Income |  |  |  | .01 | .10 | .01 |
| NR x Maternal Mental Health |  |  |  | -.18 | .18 | -.08 |
| NR x Impaired Maternal Bonding |  |  |  | -.03 | .02 | -.16 |

*Note*. We modeled pathways from all covariates to all other predictors. Covariances were allowed between predictors. ***p*<.01, ****p*<.001

## **Table S15**. Results of path models examining main and interactive effects of peripartum risk factors, including maternal perceived discrimination on social communication difficulties at age 2

|  | **Main Effects** | | | **Neighborhood Level Interactions** | | |
| --- | --- | --- | --- | --- | --- | --- |
|  | *B* | *SE* | *β* | *B* | *SE* | *β* |
| ***Covariates*** |  |  |  |  |  |  |
| *Child Age* | -.07 | .14 | -.05 | -.12 | .14 | -.09 |
| *Child Sex* | .30 | .23 | .12 | .30 | .24 | .12 |
| *Parity* | .09 | .28 | .03 | -.04 | .30 | -.02 |
| *Maternal Minoritized Racial Identity* | .42 | .48 | .17 | .55 | .54 | .21 |
| *Maternal Age (time 2)* | .01 | .02 | .05 | .02 | .03 | .09 |
| ***Main effects*** |  |  |  |  |  |  |
| Gestational Age | **-.17** | **.07** | **-.20*** | **-.19** | **.07** | **-.23**** |
| Postpartum Income | **-.46** | **.17** | **-.40**** | -.33 | .19 | -.28 |
| Maternal Mental Health | -.30 | .31 | -.10 | -.32 | .32 | -.10 |
| Impaired Maternal Bonding | **.07** | **.03** | **.29*** | **.07** | **.03** | **.26**** |
| Perceived Discrimination | -.02 | .02 | -.15 | -.02 | .02 | -.16 |
| Neighborhood Resources (NR) | -.13 | .11 | -.11 | -.21 | .12 | -.15 |
| ***Interactions*** |  |  |  |  |  |  |
| NR x Gestational Age |  |  |  | .06 | .08 | .07 |
| NR x Postpartum Income |  |  |  | **.30** | **.15** | **.21*** |
| NR x Maternal Mental Health |  |  |  | -.27 | .39 | -.10 |
| NR x Impaired Maternal Bonding |  |  |  | -.02 | .03 | -.07 |
| NR x Perceived Discrimination |  |  |  | .002 | .01 | .01 |

*Note*. We modeled pathways from all covariates to all other predictors in the models. Covariances were allowed between predictors. **p*<.05

# **Supplemental Figures**

## **Figure S1**. Flow diagram of participation across all five study time points

*Note*. To recruit the larger cohort, a medical record search identified 3,548 pregnant individuals ≥18 years receiving medical and prenatal care through the University of Pennsylvania health system between April 17, 2020, and May 1, 2020. Individuals were invited through email to participate in an online REDCap survey for the Intergenerational Exposome (IGNITE) study. Of the 3,548 individuals initially contacted, 1,173 individuals completed the online survey at time 1 (gestational age [GA], *M*=24.6 weeks, *SD*=8.4). Time 2 data were collected from the medical record system regarding childbirth experiences for 1,105 individuals who gave birth within the University of Pennsylvania health system. At time 3, we obtained postpartum survey data from *n*=823 (76% retention; *M*=11.8 weeks postpartum, SD=4.2) (Waller et al., 2022). When children were 2 years old, we started a new sub-study, the Prenatal to Preschool (P2P) study (R01MH128593; Njoroge et al., 2023), which recruited a subsample (N=251) of the original cohort who identified as White (n=129 51.4%) or Black or African American (n=122, 48.6%) (child age, M=26.43 months, SD=.95). Of the current sample of 251 participants, 61% had fully available data at all 5 time points (*n*=153), 32% had 4 time points (*n*=81) and 7% with 3 time points (*n*=17).

## **Figure S2.** Toys for observational parent-child interactions tasks

*Note*. Observational measures of child social communication/engagement from the time 5 visit were derived from qualitative and objective coding of 4 parent-child interaction tasks. First, we assessed difficulties based on *observer* ratings of child social engagement with their parent from 3 parent-child interaction tasks adapted from the Three Bags procedure, each lasting two minutes: **A)** a free play task where parent and child played freely with a dinosaur toy; **B)** a structured task where the child played with a cube shaped puzzle and the parent provided the child with any assistance; and **C)** a storybook reading task where parents were asked to read a wordless book with their child. Second, we derived an objective metric of social communication based on child expressed language. This rating was based on child language expressed across the adapted Three Bags procedure tasks (A-C) and an additional **D)** clean-up task (parents and children were asked to play with a farmer’s market basket toy and then to return all items to the basket).

## **Figure S3.** Multi-method social communication difficulties latent factor

*Note*. The multi-method social communication difficulties factor showed excellent fit (χ^2^(1)=1.04; CFI=.999; TLI=.996; RMSEA=.012). To improve model fit, self-report measures were allowed to correlate. ***p*<.01, ****p*<.001

## **Figure S4.** Distribution of child social communication difficulty factor scores at age 2


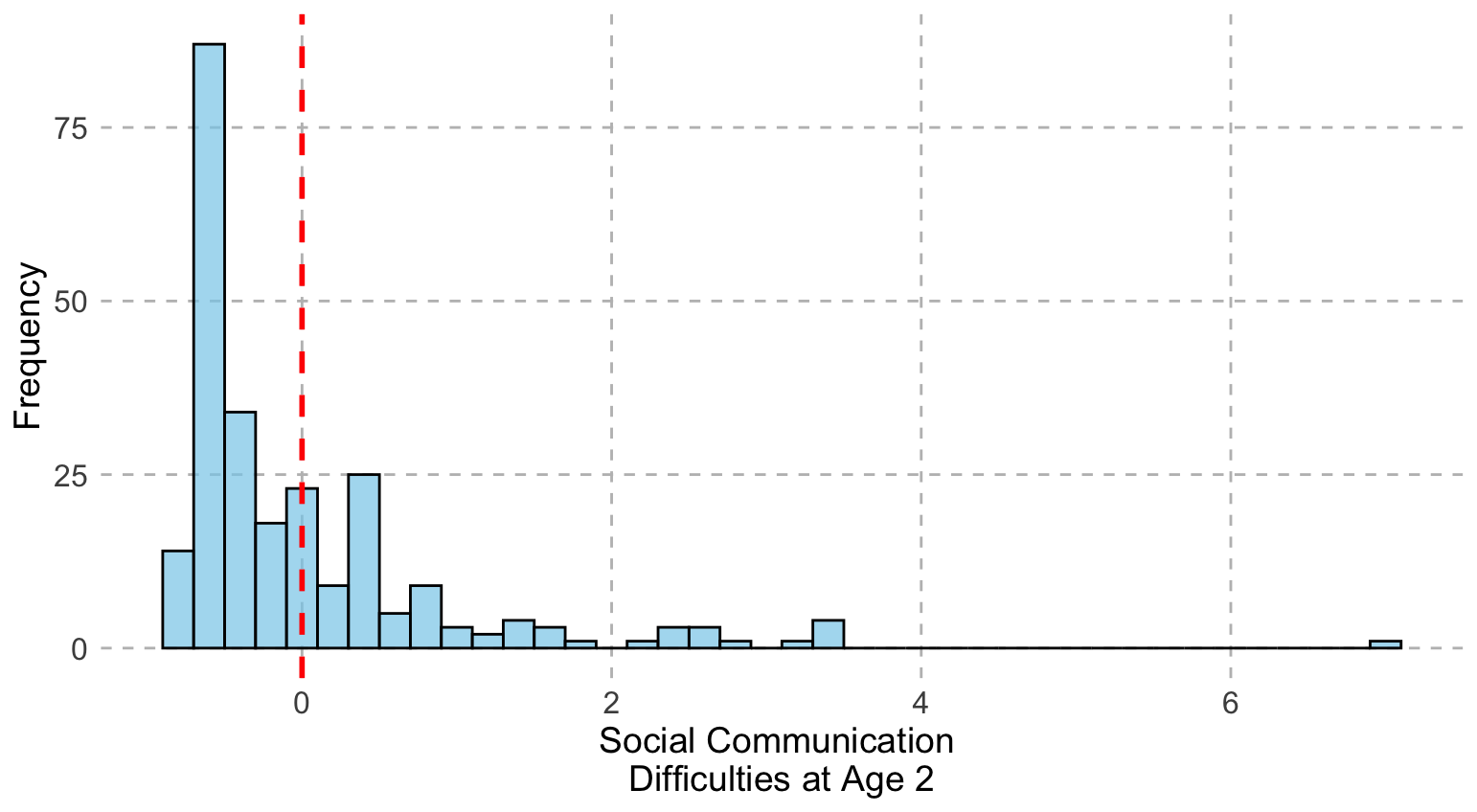


*Note*. The multi-method social communication difficulties factor showed excellent model fit (χ2(1)=1.04; CFI=.999; TLI=.996; RMSEA=.01). Social communication difficulty scores had a mean of zero (red dotted line) and a standard deviation of 0.98 (*min*=-.72, *max*=6.92) .

## **Figure S5.** Lower gestational age, household income, and impaired maternal bonding were related to greater social communication difficulties at age 2


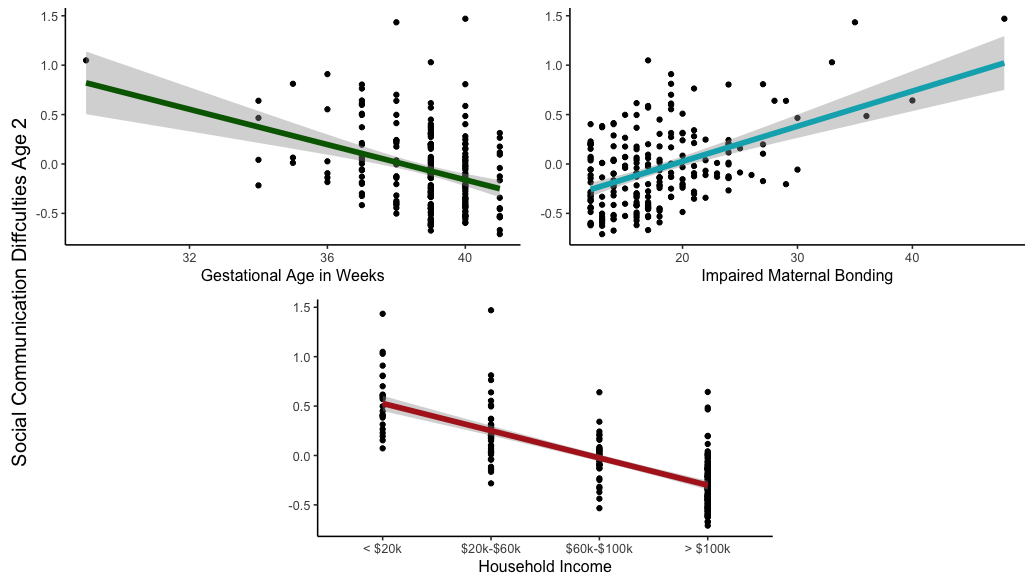


*Note*. Social communication difficulties were greatest for children born at a lower gestational age (β=-.19, *p*<.05) and for children’s whose mother’s reported lower household income (β=-.39, *p*<.01) and higher impaired maternal bonding (β=-.27, *p*<.05) in the postpartum period.
